# Inhibition of TGF-β signaling, invasion, and growth of cutaneous squamous cell carcinoma by PLX8394

**DOI:** 10.1038/s41388-023-02863-8

**Published:** 2023-10-20

**Authors:** Elina Siljamäki, Pilvi Riihilä, Ujjwal Suwal, Liisa Nissinen, Pekka Rappu, Markku Kallajoki, Veli-Matti Kähäri, Jyrki Heino

**Affiliations:** 1 MediCity Research Laboratory, University of Turku, Tykistökatu 6A, FI-20520 Turku, Finland; 2 Department of Life Technologies and InFLAMES Research Flagship, University of Turku, FI-20014 Turku, Finland; 3 Department of Dermatology, University of Turku and Turku University Hospital, Hämeentie 11 TE6, FI-20520 Turku, Finland; 4 FICAN West Cancer Research Laboratory, University of Turku and Turku University Hospital, Kiinamyllynkatu 10, FI-20520 Turku, Finland; 5 Department of Pathology, University of Turku and Turku University Hospital, Kiinamyllynkatu 10, FI-20520 Turku, Finland

**Keywords:** Squamous cell carcinoma, Growth factor signalling

## Abstract

Cutaneous squamous cell carcinoma (cSCC) is the most common metastatic skin cancer. The prognosis of patients with metastatic cSCC is poor emphasizing the need for new therapies. We have previously reported that the activation of Ras/MEK/ERK1/2 and transforming growth factor β (TGF-β)/Smad2 signaling in transformed keratinocytes and cSCC cells leads to increased accumulation of laminin-332 and accelerated invasion. Here, we show that the next-generation B-Raf inhibitor PLX8394 blocks TGF-β signaling in ras-transformed metastatic epidermal keratinocytes (RT3 cells) harboring wild-type B-Raf and hyperactive Ras. PLX8394 decreased phosphorylation of TGF-β receptor II and Smad2, as well as p38 activity, MMP-1 and MMP-13 synthesis, and laminin-332 accumulation. PLX8394 significantly inhibited the growth of human cSCC tumors and in vivo collagen degradation in xenograft model. In conclusion, our data indicate that PLX8394 inhibits several serine-threonine kinases in malignantly transformed human keratinocytes and cSCC cells and inhibits cSCC invasion and tumor growth in vitro and in vivo. We identify PLX8394 as a potential therapeutic compound for advanced human cSCC.

## Introduction

Epidermal keratinocyte-derived cutaneous squamous cell carcinoma (cSCC) is the most common metastatic skin cancer with increasing incidence worldwide [1, 2]. The molecular basis of cSCC progression from premalignant lesion, actinic keratosis, to cSCC in situ and finally to invasive cSCC is incompletely understood [2], and the prognosis of patients with advanced disease is poor [3, 4].

Laminin-332 is a major protein component in cutaneous basement membrane, and in many cancers, including cSCC, its high expression level correlates with tumor invasiveness [5–7]. We have recently shown that hyperactive H-Ras and stromal fibroblast-related induction in TGF-β signaling co-operate in the synthesis of laminin-332 in cSCC cells, leading to enhanced cancer cell invasion [8]. H-Ras is mutated in ~10% of cSCCs [9–12]. Mitogen-activated protein kinases (MAPKs) can also be activated by other mechanisms. Recently, a meta-analysis of cSCC driver mutations was performed and 30 genes were appointed to operate in signaling pathways known to be disrupted in cSCC [11]. Mutations that activate the MAPK and/or phosphoinositide 3-kinase (PI3K) pathways occurred in 31% of the tumors [11]. Accumulation of laminin-332 can be prevented by inhibition of either Ras or TGF-β signaling, which consequently decrease cSCC invasion [8]. The TGF-β signaling pathway may also promote the invasion of cSCC via activation of matrix metalloproteinase (MMP) production [13].

These previous observations encouraged us to search for new strategies for blocking the activation of Ras and TGF-β signaling pathways in cSCC. Ras is known to act via activation of ERK1/2 MAPK pathway, composed of MAPK kinase kinases (Rafs), MAPK kinases (MEKs), and MAPKs (ERKs). B-Raf inhibitors, such as dabrafenib and vemurafenib, are used in the treatment of ^V600E^B-Raf positive cancers, including metastatic melanoma and non-small-cell lung cancer [14–16] alone or in combination with a MEK1/2 inhibitor (trametinib). Undesirably, dabrafenib and vemurafenib can activate ERK1/2 pathway in cells that exhibit high Ras activity [17–19]. Recently, next-generation Raf inhibitors have been developed. PLX7904 and its optimized analog PLX8394 do not elicit paradoxical ERK1/2 activation [20–22]. PLX8394 has been shown to suppress the growth of B-Raf mutant lung cancer [23] and melanoma in vivo [24]. PLX8394 is currently in phase II clinical trial in B-Raf mutated cancers (clinicaltrials.gov NCT No: NCT02428712). PLX8394 inhibits ^V600E^B-Raf in low nanomolar concentrations, but in previously published in vivo mouse experiments after oral administration of 150 mg/kg, its serum concentrations have been high (Cmax: 164 μM after 1 h; T1/2: 3.5 h) without obvious toxicity [23].

Here, we have used three-dimensional spheroid cultures of transformed RT3 keratinocytes which harbor wild-type B-Raf and hyperactive Ras, and spheroid cocultures of RT3 cells and skin fibroblasts. We show that low micromolar concentrations of PLX8394, previously shown to be non-toxic in in vivo experiments [23] inhibit phosphorylation of Smad2 and TGF-β Receptor II (TGFβRII) and p38 activation. Consequently, PLX8394 decreased MMP-1 and MMP-13 expression and laminin-332 accumulation by RT3 cells and inhibited RT3 cell invasion. Orally administered PLX8394 inhibited growth and invasion of human cSCC in vivo in xenograft model. Thus, we identify novel serine-threonine kinase targets for PLX8394 that can be blocked by low micromolar concentrations. In general, our results suggest that serine-threonine kinase inhibitors with optimal target spectrum can be potential new therapeutic compounds for advanced cSCC.

## Results

### Serine-threonine kinase PLX8394 inhibits laminin-332 expression in transformed keratinocytes

We have previously reported accumulation of laminin-332 in the invasive front of human cSCC tumors [8]. In this study, we used spontaneously immortalized, nontumorigenic human keratinocyte cell line (HaCaT) and its H-Ras-transformed metastatic derivative RT3. These cells have been widely used to study the progression of cSCC from benign lesions to malignant tumors [25–27] and they show high constitutive activation of ERK1/2 [28]. We showed that H-Ras knockdown decreases laminin-332 expression in RT3 cells when they are cultured as 3D spheroids [8]. Furthermore, in in vitro assays laminin-332 expression correlated with cell invasion [8].

**Fig. 1:**
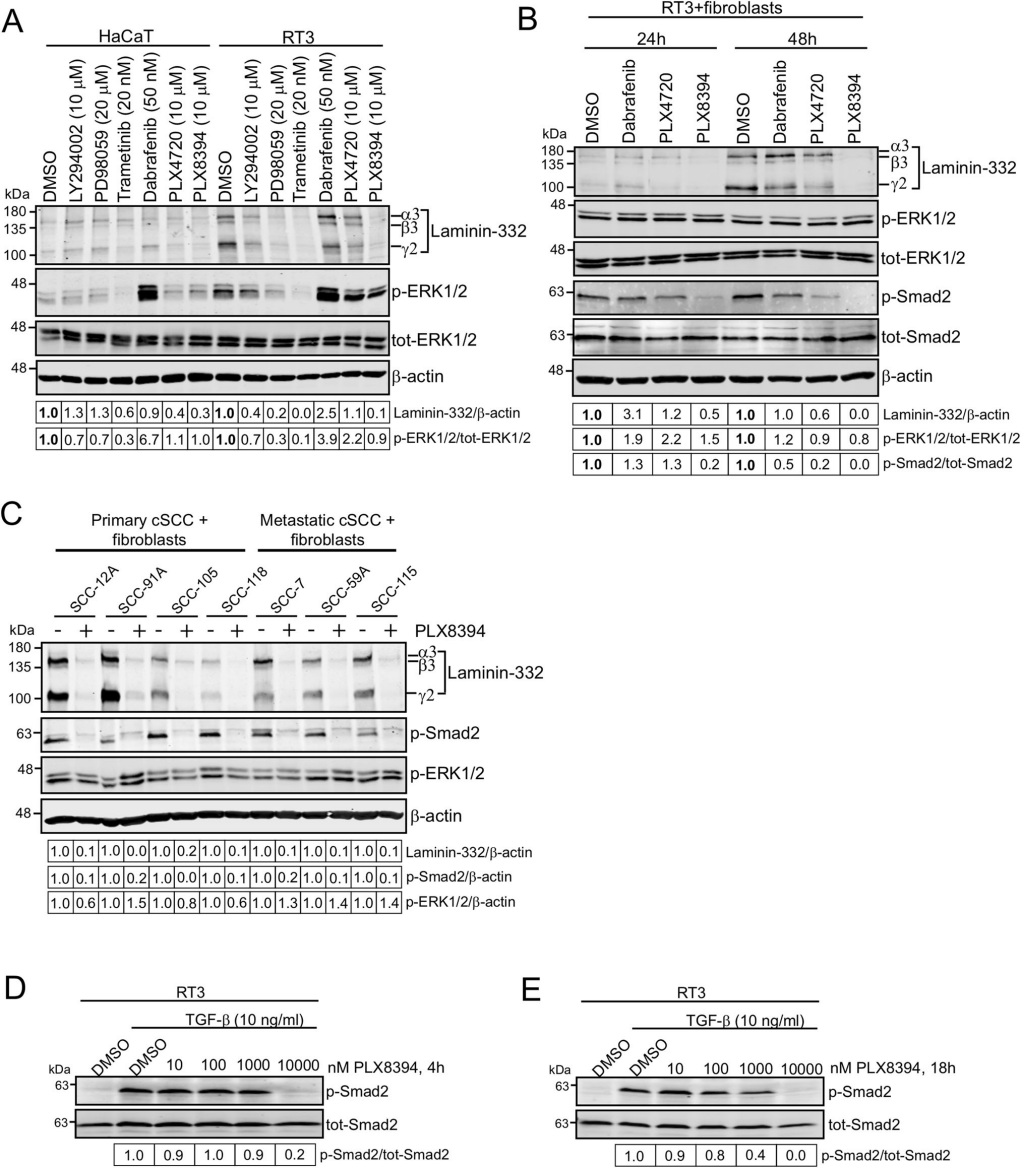
PLX8394 decreases laminin-332 synthesis and inhibits Smad2 phosphorylation both in 3D spheroids and in 2D monolayer cultures in RT3 cells. See also Supplementary Fig. S1. **A** HaCaT and RT3 cells were treated with a panel of inhibitors for 24 h in 2D monolayer conditions, followed by 3D spheroid formation. The inhibitors (LY294002 (PI3K inhibitor); PD98059, trametinib (MEK inhibitors); dabrafenib, PLX4720, PLX8394 (BRAF inhibitors) were added daily for 3 days. Laminin-332, phosphorylated ERK1/2 (p-ERK1/2), and total ERK1/2 (tot-ERK1/2) levels were analyzed by western blotting. β-actin was used as a loading control. Densitometric quantitation of laminin-332 and p-ERK1/2 levels corrected for β-actin or tot-ERK1/2 is shown below the blots. Values are relative to the levels (1.0) of DMSO control in HaCaT or RT3 cells. **B** RT3 cells were treated with dabrafenib (50 nM), PLX4720 (10 μM), or PLX8394 (10 μM) for 24 h in 2D monolayer conditions, followed by 3D spheroid formation with skin primary fibroblasts. The spheroids were grown for 24 h or 48 h before harvesting for western blotting. The levels of laminin-332, p-ERK1/2, tot-ERK1/2, p-Smad2, and tot-Smad2 were analyzed by western blotting. β-actin was used as a loading control. Densitometric quantitation of laminin-332, p-ERK1/2, and p-Smad2 levels corrected for β-actin, tot-ERK1/2, or tot-Smad2 is shown below the blots. Values are relative to the levels (1.0) of DMSO control in 24 h or 48 h samples. **C** A panel of primary (UT-SCC-12A, UT-SCC-91A, UT-SCC-105, UT-SCC-118) and metastatic (UT-SCC-7, UT-SCC-59A, UT-SCC-115) cSCC cell lines were treated with 10 μM PLX8394 for 24 h in 2D condition, followed by spheroid formation with skin primary fibroblasts. The spheroids were grown 3 days. The levels of laminin-332, p-Smad2, and p-ERK1/2 were analyzed by western blotting. β-actin was used as a loading control. Densitometric quantitation of laminin-332, p-Smad2, and p-ERK1/2 levels corrected for β-actin, tot-Smad2, or tot-ERK1/2 is shown below the blots. Values are relative to the levels (1.0) of control samples (without PLX8394) of each cell line. RT3 cells were treated with increasing concentrations of PLX8394 for 4 h (**D**) or 18 h (**E**). The cells were then subjected to TGF-β (10 ng/ml, 30 min) and harvested for western blotting. p-Smad2 and tot-Smad2 levels were analyzed by western blotting. Densitometric quantitation of p-Smad2 levels corrected for tot-Smad2 is shown below the blots. Values are relative to the level (1.0) of DMSO plus TGF-β treated samples.

Based on these previous observations, we now tested chemical inhibitors for signaling proteins in the ERK1/2 pathway, i.e. downstream effectors of Ras. Laminin-332 expression was determined by western blotting. HaCaT and RT3 cells were treated with the inhibitors for 24 h in monolayers before they were allowed to form 3D spheroids. The spheroids were allowed to grow for 72 h and DMSO treated cells were used as controls. In accordance with our previous results [8], western blotting showed that HaCaT cells do not express significant amounts of laminin-332, while this laminin is actively produced by RT3 cells (Fig. 1A). Treatment of RT3 cells with MEK inhibitors PD98059 (20 μM) and trametinib (20 nM) decreased ERK1/2 activation and consequently also laminin-332 synthesis (Fig. 1A). This is in accordance with our previous results showing that the inhibition of ERK1/2 activation decreases laminin-332 expression [8]. In accordance with previous reports [29, 30], Raf inhibitor dabrafenib (50 nM) caused paradoxical MAPK pathway activation, observed as increased (about 4-fold) ERK1/2 phosphorylation. Concomitantly, dabrafenib increased laminin-332 synthesis (Fig. 1A). Interestingly, ^V600E^B-Raf inhibitor PLX4720 (10 μM) decreased laminin-332 γ2 chain expression (~50%) when compared to DMSO treated control sample (Fig. 1A) and a potent inhibitory effect of total laminin-332 protein (~90%) was detected with next-generation Raf inhibitor PLX8394 (10 μM). PLX8394 treatment did not lead to paradoxical increase in phosphorylated ERK1/2 level, instead we observed a minor decrease (~10%) in ERK1/2 phosphorylation, which could not explain the notable downregulation in laminin-332 synthesis (Fig. 1A).

### PLX8394 inhibits TGF-β signaling both in 3D spheroids and in 2D monolayer cultures of RT3 cells

Since the significant decrease in laminin-332 expression in RT3 cells after PLX4720 and especially after PLX8394 treatment was not associated with similar inhibition in ERK1/2 phosphorylation, we analyzed their effects on the activity of the Smad signaling pathway. To activate TGF-β signaling pathway, RT3 cells were cocultured with primary human dermal fibroblasts. RT3 cells were treated with PLX8394 (10 μM) or PLX4720 (10 μM), while dabrafenib (50 nM) was used as a control. The cells were treated with the inhibitors 24 h in monolayers before spheroid formation with primary skin fibroblasts and the spheroids were allowed to grow for 24 h and 48 h. At 48 h time point western blotting showed a decrease in the phosphorylation of Smad2 after both PLX4720 and PLX8394 treatment (Fig. 1B). However, PLX8394 was clearly more potent inhibitor of Smad2 activation and it also decreased SMAD2 phosphorylation (~80%) in 24 h time point. The most potent inhibition of Smad2 phosphorylation and laminin-332 accumulation was observed after 48 h (Fig. 1B). PLX8394 slightly decreased ERK1/2 phosphorylation at 48 h time point (Fig. 1B), but the inhibition of TGF-β signaling pathway was noted as the main mechanism, by which PLX8394 prevents laminin-332 accumulation.

To further study the effect of PLX8394 on TGF-β signaling, we treated HaCaT and RT3 cells with PLX8394 (10 μM), or with DMSO as a control, in 2D cell culture condition for 24 h. The cells were then treated with TGF-β (10 ng/ml, 30 min) and harvested for western blotting. The results showed that PLX8394 totally prevented Smad2 phosphorylation both in HaCaT and RT3 cells (Supplementary Fig. S1A). To examine whether PLX8394 could affect TGF-β induced Smad activation also in 3D spheroids, HaCaT and RT3 cells were first treated with DMSO or PLX8394 (10 μM) in 2D cell culture condition for 24 h, followed by 3D spheroid formation. Two-day-old spheroids were treated with TGF-β (10 ng/ml) for 4 h. Western blotting showed that also in spheroids, PLX8394 inhibited TGF-β induced Smad2 activation in RT3 cells (Supplementary Fig. S1B). Laminin-332 expression was decreased by PLX8394 both in 2D and 3D cultured samples (Supplementary Fig. S1A, B). To conclude, these results demonstrate that PLX8394 inhibits TGF-β signaling and concomitantly decreases laminin-332 accumulation in RT3 cells.

To verify the results obtained from RT3 cells, we treated a panel of primary and metastatic cSCC cell lines with PLX8394 for 3 days. The cells were first treated with DMSO or PLX8394 (10 μM) in 2D cell culture condition for 24 h, followed by 3D spheroid formation with primary human skin fibroblasts. The results showed that PLX8394 inhibited laminin-332 synthesis in all primary (UT-SCC-12A, UT-SCC-91A, UT-SCC-105, UT-SCC-118) and metastatic (UT-SCC-7, UT-SCC-59A, UT-SCC-115) cell lines (Fig. 1C). Phosphorylation of Smad2 was also clearly decreased in all PLX8394 treated samples compared to control, whereas phosphorylation of ERK1/2 was decreased in some of the cell lines (Fig. 1C). These results indicated that PLX8394 was a potent inhibitor of TGF-β signaling in human squamous cell carcinoma.

Next, to determine the optimal PLX8394 concentration for inhibition of Smad2 phosphorylation, RT3 cells were exposed to increasing concentrations (0–10,000 nM) of PLX8394 for 4 h or 18 h, followed by TGF-β treatment (10 ng/ml, 30 min). The results showed that 10 μM concentration of PLX8394 totally inhibited TGF-β induced Smad2 activation in RT3 cells both after 4 h (Fig. 1D) and 18 h (Fig. 1E) inhibitor treatment. Inhibition by a lower concentration of 1 μM was also evident (~10% at 4 h and ~60% at 18 h; Fig. 1D, E). In addition to RT3 cells, we tested the effects of different PLX8394 concentrations on A2058 melanoma cells which harbor ^V600E^B-Raf mutation. Western blotting showed that also in A2058 cell line, 10 μM PLX8394 concentration blocked Smad2 activation both after 4 h (Supplementary Fig. S1C) and 18 h (Supplementary Fig. S1D) of inhibitor treatment. The efficacy of the inhibitor was verified by analyzing p-ERK1/2 levels in A2058 melanoma cells exposed to different concentrations (0–10,000 nM) of PLX8394. The results showed that 100 nM concentration of PLX8394 inhibited p-ERK1/2 levels after 4 h (Supplementary Fig. S1E) and after 18 h (Supplementary Fig. S1F) of inhibitor treatment, which is in accordance with previously published results obtained with ^V600E^B-Raf mutated melanoma cell line [31]. Altogether, our results show that 1-10 μM PLX8394 inhibits Smad2 phosphorylation both in cells harboring wild-type B-Raf or ^V600E^B-Raf mutation.

### PLX8394 inhibits TGF-β signaling by affecting TGF-β type II receptor kinase activity

**Fig. 2:**
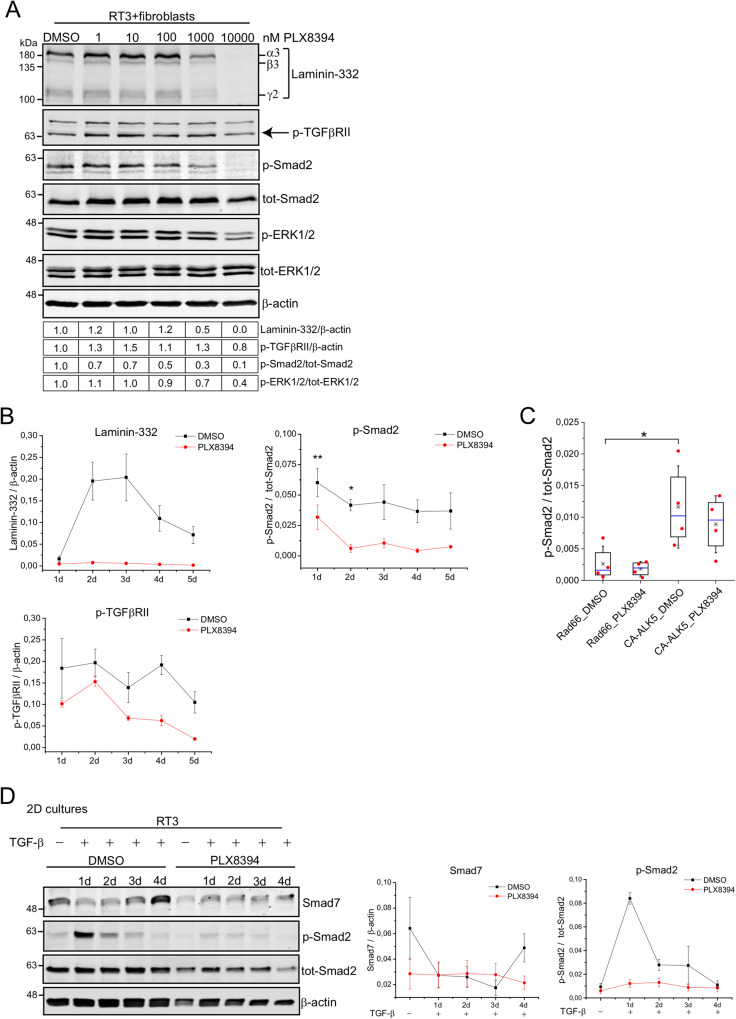
PLX8394 inhibits TGF-β signaling by affecting TGF-β type II receptor kinase activity. See also Supplementary Fig. S2. **A** RT3 cells were treated with increasing concentrations of PLX8394 for 24 h in 2D condition, followed by spheroid formation with skin primary fibroblasts. The spheroids were grown for 3 days. The levels of laminin-332, p-TGFβRII, p-Smad2, tot-Smad2, and p-ERK1/2 were analyzed by western blotting. β-actin was used as a loading control. Densitometric quantitation of laminin-332, p-TGFβRII, p-Smad2, and p-ERK1/2 levels corrected for β-actin, tot-Smad2 or tot-ERK1/2 is shown below the blots. Values are relative to the levels (1.0) of DMSO treated control samples. **B** RT3 cells were treated with 10 μM PLX8394 for 24 h in 2D condition, followed by spheroid formation with skin primary fibroblasts. The spheroids were grown for one to 5 days. The levels of laminin-332, p-Smad2, tot-Smad2 and p-TGFβRII were analyzed by western blotting. The graphs show relative protein expression to loading control from three independent biological replicates ±S.E.M. **p < 0.01, *<0.05; paired *t*-test. **C** RT3 cells were either left uninfected or infected (100 MOI) with control virus RAd66 or with adenovirus coding for constitutively active ALK5 (RAdCA-ALK5) for 48 h. The cells were then treated with 10 μM PLX8394 o/n. Phosphorylated Smad2 and tot-Smad2 levels were analyzed by western blotting. Box plots show data from four independent biological replicates (red dots), the second and third quartiles (the box), the median (blue line) and the mean (star) from all experiments ± S.D. *p < 0.05 (two-way ANOVA followed by Tukey post hoc test). **D** RT3 cells were treated with 10 µM PLX8394 and with TGF-β (10 ng/ml) in 2D condition for 1–4 days. The levels of Smad7, p-Smad2, and tot-Smad2 were analyzed by western blotting. β-actin was used as a loading control. Representative images from three independent biological replicates are shown. The graphs show relative Smad7 and p-Smad2 expression to loading control (β-actin and tot-Smad2, respectively) ± S.E.M.

A dimeric TGF-β molecule activates its downstream signaling cascades by binding to two TGF-β receptor type II proteins (TGFβRII). Consequently, TGFβRIIs activate type I receptors (TGFβRI). Both TGFβRIs and TGFβRIIs are protein serine-threonine kinases [32]. To further investigate the mechanism by which PLX8394 decreases Smad2 phosphorylation, we treated RT3 cells with increasing concentrations (0–10,000 nM) of PLX8394 for 24 h, followed by spheroid formation with primary human skin fibroblasts. The spheroids were allowed to grow for 72 h before harvesting for western blotting. The analyses showed a slight (~20%) decrease in the phosphorylation of TGFβRII after 10 μM PLX8394 treatment, along with completely inhibited Smad2 activation and laminin-332 expression (Fig. 2A). At this time point (72 h) 10 μM PLX8394 also decreased the amount of phosphorylated ERK1/2 (Fig. 2A).

To study the time-dependency of PLX8394 action, RT3 cells were treated with 10 μM PLX8394 for 24 h in monolayer culture before the formation of 3D spheroids with primary skin fibroblasts. The spheroids were grown from 1 to 5 days before harvesting for western blotting. DMSO treated control samples showed strong laminin-332 expression starting from day two (Fig. 2B). PLX8394 prevented laminin-332 expression from day 2 to 5 (Fig. 2B and Supplementary Fig. S2A). The presence of phosphorylated Smad2 was only detected in 1-day-old PLX8394 treated samples, whereas control samples showed continuous presence of phosphorylated Smad2 during the first 4 days. The experiment was repeated three times and the statistical significance of the differences was tested. The effect of PLX8394 on Smad2 phosphorylation was most evident in early time points and statistically significant on days 1 and 2 (Fig. 2B and Supplementary Fig. S2A). In accordance with the experiment shown in Fig. 2A, PLX8394 inhibited TGFβRII phosphorylation in all time points starting from day 1 (Fig. 2B and Supplementary Fig. S2A). To conclude, the results shown in Figs. 1,2, in RT3/fibroblast coculture spheroids PLX8394 completely inhibits laminin-332 production from day 2. On day 1, it is already possible to see inhibition in the phosphorylation of Smad2 and TGFβRII, indicating that in our 3D coculture model, inhibition of TGF-β signaling results in prevention of laminin-332 synthesis after 24 h of PLX8394 treatment.

Binding of TGF-β to TGFβRII leads to the recruitment and activation of TGFβRI (ALK-5), which then phosphorylates Smad2 [32]. To examine the putative role of TGFβRI in the PLX8394-related inhibition of the TGF-β signaling pathway, we used adenoviral vector coding for constitutively active mutant of ALK5 (RAdCA-ALK5) or empty vector (RAd66) as a control. RT3 cells were either left uninfected or infected for 48 h with RAdCA-ALK5 (100 MOI) or with RAd66 (100 MOI) as a control. The cells were then treated with 10 μM PLX8394 for 24 h and harvested for western blotting. As expected, RadCA-ALK5 infection increased Smad2 phosphorylation (Fig. 2C). In RadCA-ALK5-infected cells PLX8394 did not decrease p-Smad2 levels (Fig. 2C). When the cells were treated with TGF-β, it was possible to detect additional increase in Smad2 phosphorylation that could be inhibited by PLX8394 treatment (Supplementary Fig. S2B). These observations suggest that constitutively active TGFβRI (ALK-5) is not a target of PLX8394. However, we cannot exclude the possibility that PLX8394 still inhibits wild-type TGFβRI.

Finally, we analyzed the expression of Smad7, an inhibitory Smad, that attenuates intracellular TGF-β signaling through physical interaction with activated TGF-β receptor I and by blocking phosphorylation and activation of Smad2 [33]. In control samples, the strongest Smad7 expression was detected without TGF-β treatment and again in 4-day old TGF-β treated samples, which was opposite to p-Smad2 levels in control samples (Fig. 2D). In PLX8394 treated samples, Smad7 expression was weak and did not change over time. Thus, the results clearly disprove the idea that PLX8394 could regulate Smad2 phosphorylation via Smad7 activation.

### p38 MAPK regulates laminin-332 expression in RT3 cells

**Fig. 3:**
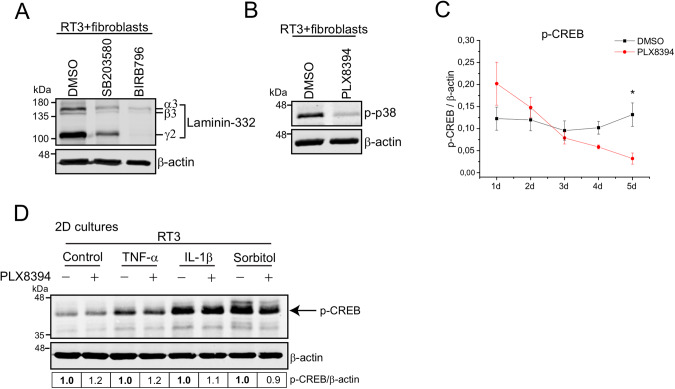
p38 MAPK regulates laminin-332 expression in RT3 cells. See also Supplementary Fig. S3. **A** RT3 cells were treated in 2D condition with p38 signaling inhibitors SB203580 (10 μM) or BIRB796 (10 μM) for 24 h, followed by 3D spheroid formation with skin primary fibroblasts. The spheroids were allowed to grow for 3 days before harvesting for western blotting. Laminin-332 level was analyzed by western blotting and β-actin was used as a loading control. Representative images from three independent biological replicates are shown. **B** RT3 cells were treated with 10 μM PLX8394 for 24 h in 2D condition, followed by spheroid formation with skin primary fibroblasts. The spheroids were grown for 3 days. The level of phosphorylated p38 (p-p38) was analyzed by western blotting and β-actin was used as a loading control. Representative images from three independent biological replicates are shown. **C** RT3 cells were treated with 10 μM PLX8394 for 24 h in 2D condition, followed by spheroid formation with skin primary fibroblasts. The spheroids were grown for 1–5 days. The level of p-CREB was analyzed by western blotting. The graph shows relative p-CREB expression to β-actin from three independent biological replicates ± S.E.M. *<0.05; paired *t*-test. **D** RT3 cells were treated in 2D condition with 10 μM PLX8394 for 24 h, followed by treatments with p38 signaling activators TNF-α (10 ng/ml, 30 min), IL-1β (10 ng/ml, 30 min) and sorbitol (400 mM, 2 h). The level of p-CREB (arrow) was analyzed by western blotting and β-actin was used as a loading control. Densitometric quantitation of p-CREB levels corrected for β-actin is shown below the blots. Values are relative to the levels (1.0) of control samples (without PLX8394) of each activator.

Smad proteins are the main mediators of TGF-β related effects. However, in addition to the Smads, TGF-βs also activate non-Smad signaling pathways, including p38 MAPKs [13, 34]. To test whether p38 MAPK pathway participates in laminin-332 synthesis in RT3 cells, we treated cells with p38 inhibitors SB203580 or BIRB796 for 24 h in monolayer cultures before they were allowed to form 3D spheroids with primary human skin fibroblasts. The spheroids were grown for 72 h. Western blotting showed that both inhibitors significantly decreased the expression of β3- and γ2-chains of laminin-332 (Fig. 3A and Supplementary Fig. S3A). Thus, in our model system laminin-332 synthesis is also regulated by p38 MAPK pathway.

We next tested whether PLX8394 is able to inhibit p38 pathway. In this purpose, we first analyzed the phosphorylation of p38. RT3 cells were treated with 10 μM PLX8394 for 24 h in monolayer culture prior to formation of 3D spheroids with primary human skin fibroblasts. The western blot results demonstrated that PLX8394 decreased phosphorylation of p38 in 3-day-old spheroids (Fig. 3B and Supplementary Fig. S3B). To further analyze PLX8394 participation in p38 signaling inhibition, we examined the phosphorylation of CREB, a down-stream target of p38. Cocultured spheroids were grown for 1–5 days before harvesting for western blotting. Spheroids grown for 3 days showed a slight decrease in CREB phosphorylation, and the phosphorylation was significantly decreased at day 5 (Fig. 3C and Supplementary Fig. S3C). This confirms that PLX8394 inhibits p38 pathway starting from day 3. To conclude, p38 pathway is yet another regulator of laminin-332 expression and the inhibition of this signaling mechanism by PLX8394 may affect laminin-332 accumulation in RT3/fibroblast spheroid model, at least in late time points.

To further study PLX8394 as an inhibitor of p38 pathway, we treated the cells with inflammatory cytokines and osmotic stress, all known activators of p38. Monolayer cultures of RT3 cells were treated with tumor necrosis factor α (TNF-α, 10 ng/ml, 30 min), interleukin 1β (IL-1β, 10 ng/ml, 30 min), and sorbitol (400 mM, 1 h). The activity of p38 was analyzed by western blotting of p-CREB (Fig. 3D). All three treatments increased CREB phosphorylation, but PLX8394 could only slightly inhibit p38 activation after sorbitol treatment and had no effect on CREB activation by TNF-α or IL-1β (Fig. 3D). Thus, PLX8394 does not appear to be a universal inhibitor of p38 pathway.

### PLX8394 inhibits RT3 cell invasion through collagen I

**Fig. 4:**
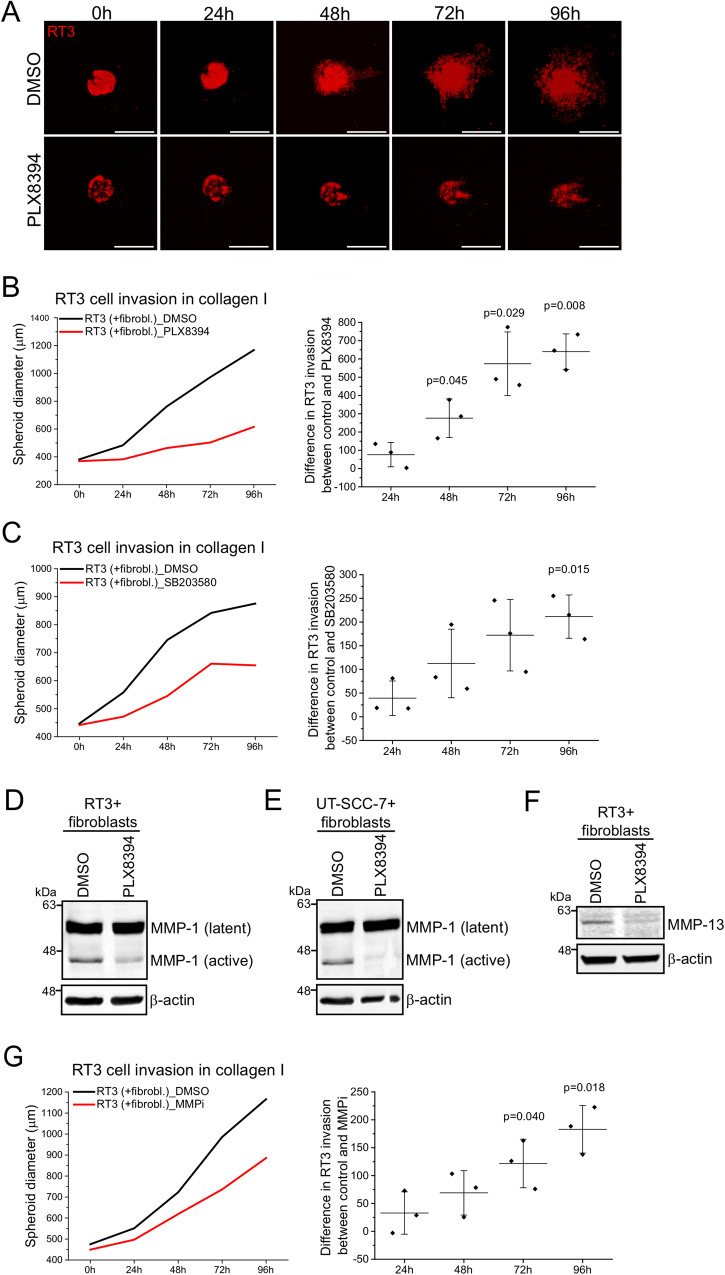
PLX8394 inhibits RT3 cell invasion through collagen I. See also Supplementary Fig. S4. **A** RT3 cells were treated with 10 μM PLX8394 for 24 h in 2D condition, followed by spheroid formation with skin primary fibroblasts. The spheroids were allowed to grow for 3 days, after which they were transferred to a 96-well plate and embedded with a collagen I gel. The invasion was followed by a confocal microscope every 24 h during 5 days. RT3 cells, red. Scale bar, 500 μm. From each time point, 2–4 spheroids were imaged and analyzed. Three independent biological replicates were performed. **B** RT3 cell invasion from cocultured spheroids that were treated as in (A). *Left*, a representative graph from three biological replicates. *Right*, analysis of RT3 cell invasion from cocultured spheroids. The graph shows the difference in RT3 cell invasion between DMSO treated control samples and PLX8394 treated samples. The graph shows mean from three independent biological replicates (squares) ± SD. (Each replicate contained 3-4 spheroids). The p values are from paired *t*-test. **C** RT3 cells were treated with 10 μM SB203580 for 24 h in 2D condition, followed by spheroid formation with skin primary fibroblasts. The spheroids were allowed to grow for 3 days, after which they were transferred to a 96-well plate and embedded with a collagen I gel. The invasion was followed by a confocal microscope every 24 h during 5 days. *Left*, a representative graph from three biological replicates. *Right*, analysis of RT3 cell invasion from cocultured spheroids. The graph shows difference in RT3 cell invasion between DMSO treated control samples and SB203580 treated samples. The graph shows mean from three independent biological replicates (squares) ± SD. (Each replicate contained 3-4 spheroids). The *p* value is from paired *t*-test. RT3 cells and cSCC cells were treated in 2D condition with PLX8394 (10 μM) for 24 h, followed by 3D spheroid formation with skin primary fibroblasts. The spheroids were allowed to grow for 3 days before harvesting for western blotting. MMP-1 (**D** and **E**) and MMP-13 (**F**) levels were analyzed by western blotting and β-actin was used as a loading control. Representative images from three independent biological replicates are shown. **G** RT3 cells were treated with 1 μM MMP Inhibitor III for 24 h in 2D condition, followed by spheroid formation with skin primary fibroblasts. The spheroids were allowed to grow for 3 days, after which they were transferred to a 96-well plate and embedded with a collagen I gel. The invasion was followed by a confocal microscope every 24 h during 5 days. *Left*, a representative graph from three biological replicates. *Right*, analysis of RT3 cell invasion from cocultured spheroids. The graph shows difference in RT3 cell invasion between DMSO treated control samples and MMP inhibitor treated samples. The graph shows mean from three independent biological replicates (squares) ± SD. (Each replicate contained 5–8 spheroids). The *p* values are from paired *t*-test.

To test whether PLX8394 affects cell invasion, we treated RT3 cells with 10 μM PLX8394 for 24 h prior to spheroid formation with skin primary fibroblasts. Three-day-old spheroids were embedded in collagen I and the invasion was followed by confocal microscope every 24 h during 5 days. Confocal images of the spheroids showed that RT3 cells treated with PLX8394 were not able to invade as efficiently as control cells that were treated with DMSO (Fig. 4A). Quantification showed that treatment with PLX8394 significantly decreased RT3 cell invasion out of coculture spheroids already after 48 h when compared to DMSO treated control samples (Fig. 4B). Fibroblast invasion from the same spheroids was not affected by PLX8394 treatment (Supplementary Fig. S4A). Western blotting of the same samples that were used in invasion assay showed that the inhibitor treatment significantly reduced p-Smad2 levels and laminin α3, β3, and γ2 chain synthesis (Supplementary Fig. S4B and C).

We also tested whether the inhibition of p38 signaling would affect RT3 cell invasion in collagen I. RT3 cells were treated with 10 μM SB203580 for 24 h prior to spheroid formation with primary human skin fibroblasts. Three-day-old spheroids were embedded in collagen I and the invasion was followed by confocal microscope every 24 h during 5 days. Quantification of images showed that treatment with SB203580 significantly decreased RT3 cell invasion out of the cocultured spheroids when compared to control samples (Fig. 4C). Fibroblast invasion was not affected by SB203580 treatment of RT3 cells when compared to DMSO treated control samples (Supplementary Fig. S4D). Accordingly, confocal images indicated that SB203580 decreased RT3 cell invasion out of the cocultured spheroids (Supplementary Fig. S4E). Western blotting of the same samples that were used in invasion assay confirmed that SB203580 decreased phosphorylation of p38 downstream target CREB (Supplementary Fig. S4F).

### PLX8394 attenuates production of MMP-1 and MMP-13 by cSCC cells

Matrix metalloproteinases (MMPs) are a family of zinc-dependent proteolytic enzymes which play a central role in tumor invasion and metastasis by degrading ECM components and other substrates, such as cytokines and growth factors [35–38]. Previously, it has been shown that the TGF-β-activated p38 pathway promotes cSCC invasion by increasing synthesis of two collagenases, MMP-1 and MMP-13 [13, 39, 40]. Since our results showed that PLX8394 was able to inhibit the fibroblast-induced TGF-β pathway, we next tested whether also MMP-1 and MMP-13 expression would be attenuated by PLX8394. RT3 and a metastatic cSCC cell line (UT-SCC-7) were treated with 10 μM PLX8394 for 24 h in monolayers, followed by spheroid formation with skin primary fibroblasts. Three-day-old spheroids were subjected to RNA extraction or harvested for western blotting. At protein level, PLX8394 significantly decreased the amount of active MMP-1 both in spheroids containing RT3 cells and primary human skin fibroblasts (Fig. 4D and Supplementary Fig. S4G), and in spheroids containing cSCC cells and human primary fibroblasts (Fig. 4E and Supplementary Fig. S4H). MMP-13 production was potently decreased in PLX8394 treated RT3 cells (Fig. 4F and Supplementary Fig. S4I). Accordingly, at mRNA level, PLX8394 treatment decreased MMP-1 and MMP-13 expression in cocultured spheroids containing RT3 cells and fibroblasts (Supplementary Fig. S4J). Furthermore, MMP-10 expression was notably downregulated in cocultured spheroids (Supplementary Fig. S4J). MMP-10 is expressed by tumor cells in cSCC [41] and it has been shown to contribute to cSCC progression by activating latent MMP-1 and MMP-13 [38, 42]. In cSCC cells, MMP-13 was downregulated in cocultured, PLX8394 treated spheroids (Supplementary Fig. S4K).

In addition, we analyzed MMP-1 and MMP-13 production in the panel of primary and metastatic cSCC cell lines that were subjected to PLX8394 for 3 days. The cells were first treated with DMSO or PLX8394 (10 μM) in 2D cell culture condition for 24 h, followed by 3D spheroid formation with human skin primary fibroblasts. The results showed that PLX8394 decreased MMP-1 synthesis in all primary (expect for UT-SCC-118) and metastatic cSCC cell lines (Supplementary Fig. S4L). MMP-13 was strongly produced in primary UT-SCC-12A and metastatic UT-SCC-7 cell lines, and to a lesser extent in a metastatic UT-SCC-115 cell line. Importantly, PLX8394 clearly inhibited MMP-13 synthesis in all of the cell lines where it was expressed (Supplementary Fig. S4M).

Lastly, we analyzed whether inhibition of MMP-1 and MMP-13 would affect RT3 cell invasion out of cocultured spheroids. RT3 cells were treated with 1 μM of MMP inhibitor III (targets MMP-1, -2 -3, -7, and -13) for 24 h prior to spheroid formation with skin primary fibroblasts. Three-day-old spheroids were embedded in collagen I and the invasion was followed by confocal microscope every 24 h during 5 days. The results showed that MMP inhibition significantly decreased RT3 cell invasion (Fig. 4G). Fibroblast invasion was only subtly delayed in MMP-inhibited spheroids (Supplementary Fig. S4N).

To conclude, we show that PLX8394 inhibits cSCC invasion in vitro by targeting both Smad2 and p38 pathways and consequently MMP-1 and MMP-13 production. PLX8394 is a more potent inhibitor of RT3 cell invasion than MMP inhibitor III, also suggesting that PLX8394 inhibits invasion by several different mechanisms. Accordingly, we have previously shown that laminin receptors and laminin-332 itself promote RT3 cell invasion inside collagen [8].

### PLX8394 inhibits the growth of human cSCC xenografts and prevents the degradation of stromal collagen in cSCC tumors

**Fig. 5:**
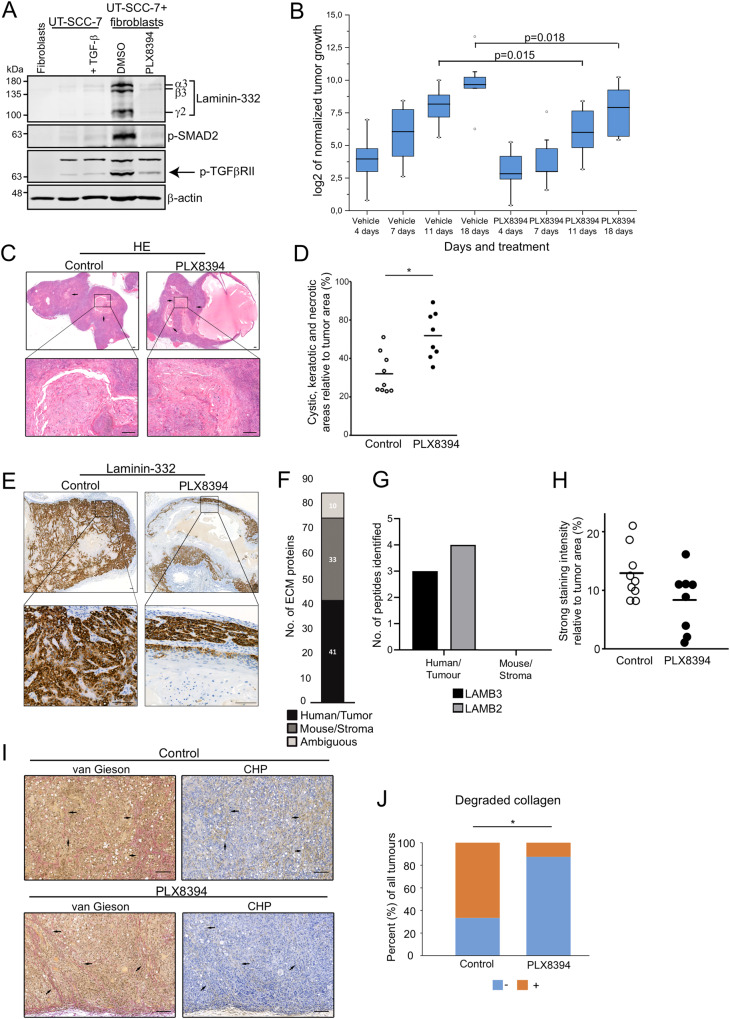
PLX8394 inhibits the growth of human cSCC xenografts and prevents the degradation of stromal collagen in cSCC tumors. See also Supplementary Fig. S5. **A** UT-SCC-7 cells were either monocultured as 3D spheroids for 24 h, followed by TGF-β treatment (5 ng/ml) for additional 48 h, or treated with 10 μM PLX8394 for 24 h in 2D condition, followed by spheroid formation with primary human skin fibroblasts and the spheroids were allowed to grow for 3 days. Fibroblasts were monocultured as 3D spheroids for 3 days and harvested for western blotting. The levels of laminin-332, p-Smad2, and p-TGFβRII (arrow) were analyzed by western blotting. β-actin was used as a loading control. Representative images from three independent biological replicates are shown. **B** UT-SCC-7 cells (5 × 10^6^) were implanted subcutaneously into the back of SCID/SCID mice, and the mice were then fed by oral gavage with PLX8394 (150 mg/kg; *n* = 8 mice) and the control mice were fed with vehicle (*n* = 9 mice) once daily for 18 days. The tumor sizes were normalized to their initial sizes and log2-transformed. The *p* values are from Student’s *t*-test. **C** Control (*n* = 9) and PLX8394 (*n* = 8) xenograft tumors were stained with haematoxylin and eosin (HE). Histological analysis of the xenograft tumors revealed different growth pattern and viability of cSCC cells in PLX8394 xenograft tumors (right panel) compared to control tumors (left panel). **D** The cystic, necrotic and keratotic areas were determined using QuPath digital image analysis software version v0.2.3 and compared to tumor size. In PLX8394 tumors, there were more fibrinotic and keratotic areas, and cystic cavities were larger compared to control tumors. Arrows indicate fibrinotic, keratotic, and cystic areas. Scale bars, 200 μm. *p < 0.05. (Mann-Whitney *U* test). **E** Expression of laminin-332 γ2 chain was examined with immunohistochemistry in human cSCC xenograft tumors of untreated control (*n* = 9, left panel) and PLX8394 treated (*n* = 8, right panel) SCID mice. Scale bars, 100 µm. **F**, **G** Tissue sections of human cSCC tumor xenograft grown in mouse were dissected with laser capture microdissection (LCM). The tissue samples were decellularized with sonication followed by reduction and alkylation of proteins. The resultant proteins were digested using an s-trap column and the peptides were analyzed by liquid chromatography tandem mass spectrometry. The identified proteins were filtered for matrisome proteins and classified based on tumor/human origin and stromal/mouse origin. **F** Number of ECM proteins identified based on tissue source in the tumor microenvironment of cSCC xenograft. **G** Number of peptides of laminin-332 identified as tumor/human or stromal/mouse origin. **H** The area of strong staining intensity of laminin-332 γ2 chain was determined digitally using QuPath bioimage analysis software version v0.2.3 and compared to total tumor area excluding large necrotic and keratotic areas. **I** Control and PLX8394 tumors were stained with van Gieson to visualize total collagen and with collagen hybridizing peptide (CHP) to visualize degraded triple-helical collagen. The association of van Gieson and CHP staining was analyzed in adjacent sections of the tumors (black arrows). In control tumors, van Gieson and CHP co-staining was detected in wide areas of tumor margin indicating collagen degradation (left panels). In PLX8394 tumors there were less co-stained areas indicating reduced amount of degraded collagen (right panels). **J** The co-staining of total collagen (van Gieson) and degraded collagen (CHP) was scored negative (−) if the co-staining was detected in limited areas and positive (+) if both stainings were detected in wide areas of tumor margins. *p < 0.05 by Fisher’s exact test.

Prior to in vivo xenograft experiments, we analyzed the effect of PLX8394 in a metastatic cSCC cell line (UT-SCC-7). Western blotting showed that TGF-β treatment (5 ng/ml, 48 h) increased laminin α3, β3, and γ2 chain synthesis when compared to DMSO treated samples. However, in cocultures of UT-SCC-7 cells and primary human skin fibroblasts laminin-332 accumulation was remarkably more prominent (Fig. 5A and Supplementary Fig. S5A). UT-SCC-7 cells were also treated with 10 μM PLX8394 o/n in monolayers, followed by spheroid formation with primary human skin fibroblasts and 3-day-old spheroids were harvested for western blotting. The results indicated that PLX8394 totally inhibited laminin-332 expression in spheroids (Fig. 5A and Supplementary Fig. S5A). Consistent with the results obtained with RT3 cells, also p-Smad2 and p-TGFβRII levels decreased after PLX8394 treatment (Fig. 5A and Supplementary Fig. S5A).

Next, we examined the effect of PLX8394 on growth of human cSCC xenograft tumors in vivo. Metastatic cSCC cells (UT-SCC-7) (5 × 10^6^ in 100 μl of PBS) were injected subcutaneously in the back of 6- to 7-week-old SCID/SCID mice. Tumors were allowed to form until they became palpable (7 days), at which point the mice were randomly divided into two groups. The mice were fed by oral gavage with PLX8394 (150 mg/kg) once daily (*n* = 8 mice). Control mice for PLX8394 were fed with vehicle (*n* = 9 mice). Tumors were measured twice per week, and mice were sacrificed and tumors harvested 18 days after the initiation of PLX8394 treatment. PLX8394 significantly suppressed tumor growth 11 days and 18 days after drug administration when compared to control tumors (Fig. 5B). We did not observe any toxicity in mice treated with PLX8394 (Supplementary Fig. S5B). We also analyzed the xenografts using hematoxylin and eosin stainings. PLX8394 tumors showed larger necrotic, keratotic and cystic areas compared to the control tumors (Fig. 5C), and the analysis revealed that PLX8394 significantly increased keratinization and necrosis inside the tumors (Fig. 5D). Thus, the effect of PLX8394 on tumor growth was much more prominent than at first suggested based on the measurements of tumor growth (Fig. 5B) or tumor weights (Supplementary Fig. S5C). Immunohistochemical staining of the xenografts revealed strong and widespread laminin γ2 chain expression in cSCC tumor cell cytoplasm in the invasive fronts of the control tumors (Fig. 5E, left panel). Next, we analyzed laminin expression in xenograft tumors by laser capture microdissection combined to mass spectrometry. In tissue microscope slides, cancer cell-containing areas were separated from tumor stroma and ECM-related proteins were recognized. The mass spectrometric analysis allowed us to determine which proteins originated from human cSCC cells and which ones were produced by mouse stromal cells (Fig. 5F). The results indicated that laminin β3 and γ2 chains were produced by human cSCC cells and not by cancer-associated fibroblasts or other stromal cell types (Fig. 5G). Furthermore, laminin-332 was the only laminin isoform detected in the xenografts (Supplementary Table S1), emphasizing the role of laminin-332 in guiding tumor invasion in cSCC. In contrast to control tumors, in PLX8394 treated xenografts γ2 chain expression was detected only in a narrow zone at the edge areas of the tumors (Fig. 5E, right panel). The analysis showed that PLX8394 clearly decreased the accumulation of laminin γ2 in tumors (Fig. 5H). However, no obvious differences could be detected in laminin γ2 production per cancer cell.

In order to analyze the collagen remodeling in cSCC xenograft tumors, we determined the amount of degraded collagen in xenografts. The total collagen in control and PLX8394 xenografts was analyzed with van Gieson (VG) staining (Fig. 5I). To determine the degradation of collagen, xenografts were stained with collagen hybridizing peptide (CHP), which specifically binds to degraded, unfolded triple-helical collagen. The adjacent sections of control and PLX8394 tumors were analyzed to detect the co-staining of VG and CHP (Fig. 5I). The staining was scored negative (−) if the co-staining was detected in limited areas and positive (+) if both stainings were detected in wide areas of tumor margins. In general, collagen was detected in the intercellular space of cSCC tumors in the peripheral areas of control and PLX8394 tumors (Fig. 5I). In control xenograft tumors the co-staining of VG and CHP (indicating collagen degradation) was significantly more abundantly seen throughout the tumor edges (67%) (Fig. 5J), whereas in tumors removed from PLX8394 treated mice the co-staining was detected only in limited areas (13%) (Fig. 5J). These results indicate that PLX8394 treated tumors contain less stromal degraded triple-helical collagen molecules than control tumors.

**Fig. 6:**
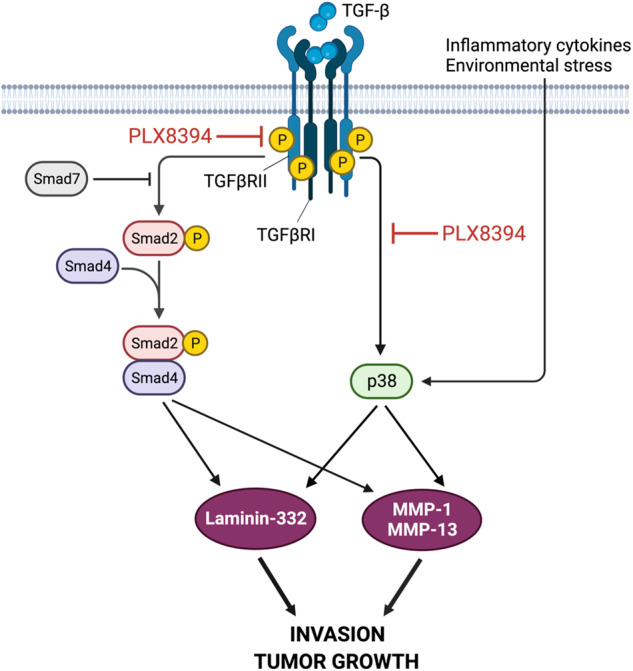
A schematic representation of serine-threonine kinase inhibition by PLX8394. PLX8394 inhibits TGF-β signaling by attenuating phosphorylation of TGFβRII and Smad2. Concurrently, PLX8394 inhibits TGF-β-induced Smad2 and p38 activation resulting in decreased MMP-1 and MMP-13 production. PLX8394 does not inhibit p38 activation induced by inflammatory cytokines or environmental stress. As a result, laminin-332 expression is downregulated leading to decreased cSCC cell invasion and tumor growth. The figure was created with BioRender.com.

To conclude, we show that in 3D spheroids containing transformed keratinocytes and skin primary fibroblasts low micromolar concentrations of PLX8394, previously shown to be non-toxic in in vivo experiments [23], inhibit Smad2 and TGFβRII phosphorylation and p38 activation. Consequently, MMP-1 and MMP-13 synthesis and laminin-332 accumulation are inhibited leading to attenuated cell invasion and tumor growth (Fig. 6). These results suggest that PLX8394 and comparable serine-threonine kinase inhibitors with optimal target spectrum can be potential new drugs for advanced and metastatic cSCC.

## Discussion

We have previously reported that laminin-332 accumulates into invasive front areas in human cSCC tumors [8]. We also named the concomitant activation of Ras and TGF-β/Smad2 pathways as one mechanism that explains this process [8]. In a recent meta-analysis, mutations that activate MAPK and/or PI3K pathways occurred in 31% of the tumors [11]. As in many cancers, also in skin cancer models TGF-β may act as either tumor suppressor or tumor promoter [43, 44]. The constitutive over-expression of TGF-β may protect suprabasal keratinocytes from hyperplasia [45]. However, at a later phase of cancer progression TGF-β often promotes invasion and metastasis [45, 46].

Here, our aim was to search for inhibitors that could block the critical signaling pathways and consequently inhibit the growth and invasion of SCC tumors. In assays based on three-dimensional spheroid cell cultures, PLX8394 was found to fulfill our requirements since, unexpectedly, it also effectively prevented the phosphorylation Smad2. PLX8394 represents the new generation ^V600E^B-Raf inhibitors, developed especially for treatment of melanoma. First-generation ^V600E^B-Raf inhibitors, such as dabrafenib and vemurafenib, had improved the treatment and prognosis of cancer patients, but the occurrence of paradoxical ERK1/2 activation limits their use. After vemurafenib treatment, most melanoma patients develop resistance within 1 year [47]. In addition, significant amount of patients treated with this Raf inhibitor developed Ras-driven cSCCs [48, 49]. PLX8394 and other next-generation Raf inhibitors are highly selective for ^V600E^B-Raf and do not cause paradoxical MAPK/ERK1/2 signaling activation [20–22]. PLX8394 inhibits ^V600E^B-Raf with low nanomolar concentrations. Still in animal experiments, orally administrated PLX8394 can reach high micromolar concentrations in serum without toxic effects [23]. In our hands, the inhibition of Smad2 phosphorylation was detected when PLX8394 was used in low micromolar concentrations. The effect was explained by the fact that PLX8394 inhibited TGFβRII, another serine-threonine kinase. We could not find evidence indicating simultaneous inhibition of TGFβRI, but we could not completely exclude this possibility either. Low micromolar concentrations of PLX8394 did not cause paradoxical activation of ERK1/2 signaling. Furthermore, we could see the inhibition of p38 MAPK pathway after PLX8394 treatment. This was obvious in later time points in spheroid cultures. The ability of PLX8394 to block p38 pathway was dependent on which activator was used. Importantly, TGF-β can also regulate p38 pathway in cSCC cells [13, 34] and in our experimental settings this is the most probable mechanism. Thus, our data indicate that when used in low micromolar concentrations, PLX8394 is not anymore specific to ^V600E^B-Raf but it is also able to inhibit other protein serine-threonine kinases effectively.

Previously, it has been shown that the TGF-β signaling-activated p38 pathway increases synthesis of two collagenases, MMP-1 and MMP-13 [13, 39, 40]. Here, in spheroids containing RT3 cells and human skin fibroblasts, PLX8394 was able to inhibit the synthesis of primary collagenolytic MMPs, collagenase-1 (MMP-1) and collagenase-3 (MMP-13), both at mRNA and protein levels. Interestingly, also the expression of MMP-7, which is associated with aggressive behavior of cSCC tumors [50] was downregulated.

PLX8394 was also shown to be an effective inhibitor of cell invasion. Our previous report connected the invasion of cSCC cells and ras-transformed keratinocytes to the accumulation of laminin-332 and the action of laminin receptors [8]. Here, we show that MMPs also participate in this process. The fact that PLX8394 was a more potent inhibitor of RT3 cell invasion than MMP inhibitor III, is in full agreement with the idea that PLX8394 inhibits invasion by both mechanisms.

Based on our results, we concluded that low micromolar concentrations of PLX8394 inhibit several serine-threonine kinases and consequently suppress signaling pathways linked to the progression of cSCC. Thus, it was justified to test PLX8394 in a mouse xenograft model utilizing human cSCC cells. In the xenograft assays, we used the human metastatic cSCC-derived cell line (UT-SCC-7). Importantly, before in vivo assays, we had confirmed that PLX8394 had similar effects in four primary and three metastatic cSCC cell lines on Smad2 activation, MMP synthesis, and laminin-332 expression as we had observed when using ras-transformed keratinocytes. Oral PLX8394 treatment significantly reduced the growth of cSCC xenografts in mice. Importantly, in the PLX8394 treated group the non-cellular, necrotic foci inside the tumors were remarkably larger. This indicates that the effect of PLX8394 on tumor growth was much more drastic than could have been estimated based on the tumor size measurements only.

Numerous studies have proposed that laminin-332 may play an important role in the progression of human SCC (reviewed in [5]). Our previously published results have indicated that in human tumors the laminin-positive areas also contain cells with phosphorylated ERK1/2 and Smad2 [8], suggesting that the molecular mechanisms observed in spheroid cultures also occur in tumors in vivo. Here, we continued this line of studies using mass spectrometric analysis of xenograft tumors. The results indicated that laminin is mainly produced by human cancer cells instead of mouse stromal cells. However, the significant effect of PLX8394 on tumor growth could not directly be linked to laminin-332 accumulation in xenografts, since also in the PLX8394 treated mice the remaining tumor cells showed positive staining for laminin γ2 chain.

We also analyzed the effects of PLX8394 on collagen remodeling in xenograft tumors. For this purpose, we used a specific peptide probe that recognizes degraded collagen. The results indicated that PLX8394 tumors contain significantly less stromal degraded triple-helical collagen molecules than control tumors. Previously, it has been documented that MMP-1 and MMP-13 expression is enhanced by TGF-β-activated p38 MAPK pathway in head and neck SCC, promoting collagenolytic and invasive capacity of the tumor cells [13, 39, 40, 51]. The essential role of MMPs has also been reported in cSCC [41, 50, 52].

To conclude, we show that in 3D spheroids containing transformed keratinocytes and skin primary fibroblasts PLX8394, used in concentrations previously proved to be non-toxic in in vivo experiments [23], inhibit Smad2 and TGFβRII phosphorylation and p38 activation. Consequently, laminin-332 accumulation and MMP synthesis are inhibited leading to attenuated cell invasion. PLX8394 also significantly prevents the growth of cSCC xenografts. In more general terms our results indicate that it is possible to find inhibitors for protein serine-threonine kinases that have a favorable target spectrum that enables the prevention of cSCC tumor growth. These observations raise the question about the potential benefits of optimal wide-spectrum inhibitors when compared to highly selective inhibition of one single proto-oncogene. Based on our results we propose PLX8394 as a promising molecule for future tests in advanced and metastatic human cSCC.

## Materials and methods

### Ethical issues

All the methods used in this study were carried out in accordance with the relevant guidelines and regulations. The experiments with mice were performed with the permission of the State Provincial Office of Southern Finland, according to institutional guidelines (ESAVI/24742/2020). The use of cSCC cell lines was approved by the Ethics Committee of the Hospital District of Southwest Finland. The research was carried out according to the Declaration of Helsinki. All the patients gave their written informed consent before the surgery.

### Cell lines and cell culture

Primary (UT-SCC-12A, UT-SCC-91A, UT-SCC-105, UT-SCC-118) and metastatic (UT-SCC-7, UT-SCC-59A, UT-SCC-115) cSCC cell lines were established from surgically removed cSCC of the skin in Turku University Hospital [53]. The spontaneously immortalized non-tumorigenic human keratinocyte-derived cell line HaCaT [25] and the H-Ras-transformed tumorigenic HaCaT cell line RT3 [26] were kindly provided by Dr. Norbert Fusenig (German Cancer Research Center, Heidelberg, Germany). A2058 melanoma cell line was a kind gift from Professor Klaus Elenius (Institute of Biomedicine, University of Turku, Finland). A2058 cell line was authenticated by short tandem repeat DNA profiling. Genotyping was performed by the Institute for Molecular Medicine Finland FIMM Technology Centre, University of Helsinki. The cSCC cell lines were authenticated by STR DNA profiling (DDC Medical, Fairfield, OH) [54]. Primary adult human skin fibroblasts were from the cell line collection of the Medical Biochemistry/the University of Turku and a kind gift from Professor Risto Penttinen. The fibroblasts were from male donors aged 19 and 24 years. The fibroblasts were used up to passage number 14. Normal human dermal fibroblasts from adult skin (C-12302) were purchased from PromoCell and they were used up to passage number 12.

All cell lines were grown in Dulbecco’s modified Eagle’s medium (DMEM with 4.5 g/L glucose; 12-614 F, Lonza) supplemented with 10% fetal calf serum (FCS), L-glutamine (6 nmol/L), penicillin (100 U/ml), and streptomycin (100 μg/ml). 1 × MEM non-essential amino acids (11140-035, Gibco) were added to cSCC culture medium. Geneticin-418 (200 μg/ml) was added to the medium of the H-Ras-transformed RT3 cell line. All cell lines were routinely tested to be negative for mycoplasma contamination using MycoAlert PLUS Mycoplasma Detection Kit (LT07-710, Lonza).

### 3D spheroid cultures

The information below on experimental parameters, spheroid preparation, and growth conditions is based on MISpheroID guidelines and recommendations [55]. For western blots and invasion assays, 3D spheroids were made in micro-molds according to the manufacturer’s instructions (MicroTissues 3D Petri Dish micro-mold spheroids, Sigma-Aldrich) with 2.5 × 10^5^ cells in one mold (monocultures; 7000 cells/spheroid) or 5.0 × 10^5^ cells in one mold (cocultures; 14 000 cells/spheroid). In cocultures, the cell ratio was 1:1 (RT3*/*cSCC cells and fibroblasts, respectively). The spheroids were grown in serum-free DMEM medium for one to 5 days at 37 °C in an incubator environment of 20% O_2_, and 5% CO_2_. Ascorbic acid (50 μg/ml) was added daily.

### Cell infection with recombinant adenoviruses

Control adenovirus RAd66 [56] was kindly provided by Dr. Gavin W.G. Wilkinson (University of Cardiff, Cardiff, UK), and constitutively active ALK5 (RAdCA-ALK5) [57] was a kind gift from Dr. Aristidis Moustakas (Ludwig Institute for Cancer Research, Uppsala, Sweden). RT3 cells were treated o/n with DMSO as a control, or with 10 μM PLX8394 in DMEM supplemented with 0.5% FCS. Next day, RT3 cells were either left uninfected or infected with RAd66 or RAdCA-ALK5 adenoviruses with MOI 100 for 6 h in DMEM supplemented with 0.5% FCS. Thereafter, the medium was replaced with fresh DMEM supplemented with 0.5% FCS and the incubations were continued for 48 h. After that, the infected cultures were treated with TGF-β (10 ng/ml) for 30 min/+37 °C, and the cells were harvested in RIPA Lysis and Extraction Buffer (89900, Thermo Fisher Scientic) for western blot analysis.

### Inhibitor, growth factor, and cytokine treatments

For inhibitor treatments, the cells were first treated in 2D condition with LY294002 (10 μM; L9908, Sigma-Aldrich), PD98059 (20 μM; 513000, Sigma-Aldrich), Trametinib (20 nM; S2673, Selleckchem), Dabrafenib (50 nM; S2807, Selleckchem), PLX4720 (10 μM; S1152, Selleckchem), PLX8394 (1-10000 nM; HY-18972, MedChemExpress), SB203580 (10 μM; 559389, Sigma Aldrich), BIRB796 (10 μM; 1358, Axon Medchem) or MMP Inhibitor III (1 μM; 444264, Sigma Aldrich). The inhibitors were diluted in DMEM supplemented with 0.5% FCS and incubated for 24 h at 37 °C. The control samples were treated with 0.1–0.01% dimethyl sulfoxide (DMSO) in DMEM supplemented with 0.5% FCS for 24 h at 37 °C. The next day, 3D spheroids were constructed either with or without skin primary fibroblasts and the spheroids were allowed to grow in serum-free DMEM for 3 days (invasion assays) or for 1–5 days (western blotting). Fresh serum-free DMEM medium supplemented with the inhibitors (or 0.1–0.01% DMSO) and ascorbic acid (50 μg/ml) was added daily. TGF-β (T7039, Sigma-Aldrich) treatment was performed to 2-day old spheroids for 4 h (10 ng/ml) or 48 h (5 ng/ml) in +37 °C and to 2D samples for 30 min (10 ng/ml) in +37 °C.

For treatments with tumor necrosis factor-α (TNF-α), interleukin-1β (IL-1β), and sorbitol, RT3 cells were first treated in 2D condition with 10 μM PLX8394 or as a control with 0.1% DMSO o/n at 37 °C. Next day, the cells were treated with TNF-α (10 ng/ml; H8916, Sigma-Aldrich) and IL-1β (10 ng/ml; 407615-5, VWR) for 30 min/+37 °C, or with D-sorbitol (400 mM, Sigma Aldrich) for 1 h/+37 °C. After that, the cells were harvested for western blotting.

### CellTracker labeling for invasion assays

RT3 cells and skin primary fibroblasts were labeled with CellTrackers in 2D condition; RT3 cells in red (CellTracker Orange CMTMR Dye, C2927, Invitrogen) and fibroblasts in green (CellTracker Green CMFDA Dye, C2925, Invitrogen). The cells were labeled with 2.5 μM dye in DMEM supplemented with 10% FCS for 1 h/+37 °C, washed twice with PBS and constructed into spheroids. After 3 days, the spheroids were used in invasion assays.

### Invasion assays from 3D spheroids in collagen I

The cells were stained with CellTrackers as described above, constructed into spheroids, and allowed to grow for 3 days. Ascorbic acid (50 μg/ml in serum-free DMEM medium) was added daily. Three-day-old spheroids were plated on collagen I coated 96-well plates (0.035 mg/ml; Collagen solution from bovine skin, C4243, Sigma) and collagen I gel (2.0 mg/ml; Type I Bovine Collagen Solution, 5010, Nutragen) was layered on the top of the spheroids. DMEM supplemented with 10% FCS was added above the collagen gel. To study the effect of PLX8394 or SB203580 on cell invasion, the medium on top of the collagen I gel was supplemented with 10 μM PLX8394 or 10 μM SB203580, and in control samples with 0.1% DMSO. To study the effect of MMP inhibition on cell invasion, the medium on top of the collagen I gel was supplemented with 1 μM MMP Inhibitor III, and in control samples with 0.01% DMSO. Spheroids were allowed to invade for 96 h and they were imaged every 24 h with Zeiss LSM880 confocal microscope. Fiji, an image processing platform based on ImageJ [58] was used to calculate the invasion, i.e., the area covered by cells. The cell invasion of 2–4 spheroids from each sample was analyzed from three independent biological replicates.

### Real-time quantitative PCR

RT3 and UT-SCC-7 cells were treated in 2D condition with 10 μM PLX8394 o/n before spheroid formation with or without skin primary fibroblasts. The spheroids were grown for 3 days and PLX8394 (10 μM) and ascorbic acid were added daily. Total RNA was extracted from the spheroids by using NucleoSpin RNA Mini kit (740955, Macherey-Nagel), and 0.25 µg of total RNA was reverse transcribed into cDNA with random hexamer (C118A, Promega) and M-MLV Reverse Transcriptase H Minus (M368B, Promega) for real-time quantitative reverse transcriptase-PCR (qRT-PCR) analysis. Specific primers and probes for *MMP1*, *MMP7*, *MMP10,* and *MMP13* (Supplementary Table S2) were designed as previously described [59]. qRT-PCR reactions were performed utilizing the QuantStudio 12 K Flex (Thermo Fisher Scientific) at the Finnish Functional Genomics Centre in Turku, Finland. qRT-PCR amplification was done using the following protocol: hold stage 2 min at 50 °C, 10 min at 95 °C, and PCR stage for 40 cycles 0.15 min at 95 °C and 1 min at 60 °C. *ACTB* (β-actin) or *GAPDH* mRNA was used as reference (Supplementary Table S2). Samples were analyzed using the standard curve method in three parallel reactions with threshold cycle values <5% of the mean threshold cycle.

### Western blot analysis

Samples were harvested in RIPA Lysis and Extraction Buffer (89900, Thermo Fisher Scientific), separated in 8–12% SDS-polyacrylamide gels, and electroblotted onto nitrocellulose membrane (sc-3718, Santa Cruz). The following antibodies were used: laminin-5 (1:1000, ab14509, Abcam); phospho-p44/42 MAPK (1:1000, #9101), p44/42 MAPK (1:1000, #9102), phospho-Smad2 (1:1000, #3108), Smad2 (1:1000, #5339), phospho-p38 MAPK (1:1000, #9211) and phospho-CREB (1:1000, #9198) (all from Cell Signaling Technology); phospho-TGFβRII (1:1000, ab183037, Abcam), Smad7 (1:1000, #42-0400, Thermo Fisher Scientific); MMP-1 (1:1000, IM35, Sigma Aldrich), MMP-13 (1:500, MAB3321, Sigma-Aldrich) and β-actin (1:50 000, A-1978, Sigma-Aldrich).

The membranes were incubated with the primary antibodies overnight at +4 °C, followed by incubation with secondary antibodies (926-32213, 926-32212, 926-68072, or 926-68073, all diluted 1:10,000, LI-COR Biosciences) for 1 h /RT. The membranes were scanned with Odyssey infrared imaging system (LI-COR), and the band intensities were determined by densitometric analysis using the Odyssey Image Studio Lite software. Average from three or four independent biological replicates was calculated.

### Laser capture microdissection and mass spectrometry

Freshly prepared 8 μm tissue sections of cutaneous squamous cell carcinoma xenograft grown in mice were placed on MembraneSlides 1.0 PEN (Carl Zeiss, Germany). The tumor area (containing human tumor and mouse stromal part) of approximately 9 × 10^6^ μm^2^ was dissected using Zeiss PALM LCM system. The laser microdissected tissue was further processed for mass spectrometry with the s-trap column as described [60]. The digested peptides (1 μg) dissolved in 0.1% formic acid were separated by reverse-phase chromatography using nanoflow HPLC system (Easy-nLC1000, Thermo Fisher Scientific) coupled to the Q Exactive HF mass spectrometer (Thermo Fisher Scientific). The peptide mixture was injected into a 15 cm C18 column (75 μm × 15 cm, ReproSil-Pur 3 μm 200 Å C18-AQ, Dr. Maisch HPLC GmbH, Ammerbuch-Entringen, Germany) and separated with a 60-min gradient (8–35% B in 50 min, 35-100% B in 2 min and held at 100% B for 8 min, where A is 0.1% formic acid and B is acetonitrile/water (95:5 (v/v)) with 0.1% formic acid.). Full scan over the m/z range 300–1750 at a resolution of 120,000 was performed followed by data-dependent acquisition at an isolation window of 2.0 m/z and dynamic exclusion time of 30 sec. The top 10 ions were fragmented by high-energy collisional dissociation and scanned over 200–2000 m/z at a resolution of 15,000.

### Proteomic data analysis

The raw tandem mass spectra were processed using Maxquant software (version 1.6.10.43) against a database containing reviewed (SwissProt) human sequences along with reviewed and unreviewed (TrEMBL) mouse sequences of UniProtKB release 2020_03. Fixed modification of carbamidomethyl (C) and variable modification of oxidation (M, P, K) were included. Peptide spectrum match and false discovery rate were set at 1%. A maximum of two missed cleavages was allowed. Protein identification was designated as valid in the sample if the protein contains at least two identified unique or razor peptides. Contaminant proteins, reverse identification, and identification by site were filtered from the identified proteins. Proteins identified were annotated using the most recent human and mouse matrisome database MatrisomeDB (downloaded in January 2021) from http://matrisomeproject.mit.edu/.

### Confocal imaging

The spheroids were imaged with Zeiss LSM880 AiryScan confocal microscope (Zeiss, Jena, Germany) (10× objective; numerical aperture [NA] 0.3; green CellTracker excitation at 488 nm, orange CellTracker excitation at 543 nm). The imaging was performed at the Cell Imaging and Cytometry Core, Turku Bioscience Centre, Turku, Finland, with the support of Biocenter Finland.

### Human cSCC xenografts

Metastatic cSCC cells (UT-SCC-7) (5 × 10^6^ in 100 μl of PBS) were injected subcutaneously into the back of 7-week-old SCID/SCID female mice (CB17/Icr-*Prkdc*^*scid*^/IcrIcoCrl) (Charles River Laboratories). Tumors were allowed to form until they became palpable (7 days), at which point the mice were randomly divided into two groups. The mice were fed by oral gavage with PLX8394 (150 mg/kg) once daily (*n* = 8 mice). The control mice were fed by oral gavage with vehicle once daily (*n* = 9 mice). PLX8394 was dissolved in polyethylene glycol 400 (PEG 400) [20% (vol/vol)], DL-α-Tocopherol methoxypolyethylene glycol succinate (TPGS-750-M) [5% (vol/vol)] and water [75% (vol/vol)] just before administration, and vortexed continuously throughout the dosing period. The vehicle was PEG 400 [20% (vol/vol)], TPGS-750-M [5% (vol/vol)], and water [75% (vol/vol)]. Tumors were measured twice per week, and mice were sacrificed and tumors harvested 18 days after the initiation of PLX8394 treatment. Tumor volume was calculated with the formula: *V* = (length × width^2^)/2 [61]. PLX8394 (HY-18972) and PEG 400 (HY-Y0873A) were obtained from MedChemExpress. TPGS-750-M (763896) was from Sigma-Aldrich. All mice used in this study were maintained in the animal facility at the Central Animal Laboratory, University of Turku in accordance with the institutional guidelines and with the permission of the State Provincial Office of Southern Finland (permit number ESAVI/24742/2020). The humane endpoint for a single tumor was 1.2 cm in any direction. At the experimental endpoint, the mice were euthanized by exposure to carbon dioxide followed by cervical dislocation.

### Immunohistochemistry (IHC)

The paraffin-embedded, formalin-fixed control and PLX8394 xenograft tumors were stained with mouse monoclonal laminin γ2 antibody (1:100; sc-28330, Santa Cruz Biotechnology Inc.) using Mayer’s haematoxylin as counterstain [62]. The primary antibody was replaced with PBS for negative control. Immunostainings were performed in Core Facilities of the Institute of Biomedicine, University of Turku as previously described [63–65]. The stained tumor slides were digitally scanned using a Pannoramic 1000 Slide Scanner (3DHistech, Budapest, Hungary). To evaluate tumor histology and laminin γ2 expression level in tissue sections whole slices of xenograft tumors were analyzed digitally by QuPath bioimage analysis software version v0.2.3 [66]. Large necrotic and keratotic areas were excluded from the total tumor area calculations. The strong immunostaining of laminin γ2 was detected digitally and compared to total tumor area. Statistical analyzes of tumor histology were performed with two-tailed Mann-Whitney U-test using SPSS Statistics software for Windows, version 28 (IBM, Armonk, NY).

Visualization of collagen was performed by staining paraffin-embedded, formalin-fixed xenograft tumors from control and PLX8394 treated mice with VG and B-CHP (BIO300, 3Helix, Salt Lake City, Utah) according to manufacturer’s instructions [67], as previously described [68]. The statistical analysis for CHP was performed with Fisher’s exact test.

### Statistical analysis

All quantitative data are presented as mean ± standard deviation or standard error of the mean as stated in the figure legends. Shapiro Wilk test was used to test normality assumption. Levene’s test was used to test the homogeneity of variances between the statistically compared groups. For quantitative studies, the amount of analyzed samples was determined by how many replicates were needed to gain a significant *p* value (under 0.05) for observed differences. For data following the normal distribution a minimum of three samples were needed for statistically significant results. Statistical differences were determined using either paired *t* test, Student’s *t* test (for dependent or independent samples) or analysis of variance complemented by appropriate post hoc tests (Tukey (if variances between the statistically compared groups were similar) or Dunnett’s T3 (if variances between statistically compared groups were not similar)). Statistical comparison of immunohistochemical data was performed using Fisher’s exact test or Mann-Whitney *U* test. Origin 2015 software (OriginLab Corporation) and SPSS (IBM Corporation, version 25) were used to perform the analyses. Only two-tailed *p* values < 0.05 were considered as statistically significant.

### Supplementary information

Supplementary Materials

## Data Availability

The mass spectrometry proteomics data have been deposited to the ProteomeXchange Consortium via the PRIDE [69] partner repository with the dataset identifier PXD033023. Further information and requests for resources and reagents should be directed to and will be fulfilled by the corresponding authors.
