# Supplementary material for: Inhibition of TGF-β signaling, invasion, and growth of cutaneous squamous cell carcinoma by PLX8394
Source: Oncogene. 2023 Oct 20;42(49):3633–47. doi: 10.1038/s41388-023-02863-8 (PMC10691969; doi:10.1038/s41388-023-02863-8)
Supplement: Supplementary file 1 — Supplementary Materials [file 41388_2023_2863_MOESM1_ESM.pdf]

Supplementary Materials for

Inhibition of TGF-β signaling, invasion and growth of cutaneous squamous cell carcinoma by PLX8394

Siljamäki *et al*.

Supplementary Figure S1, related to Figure 1

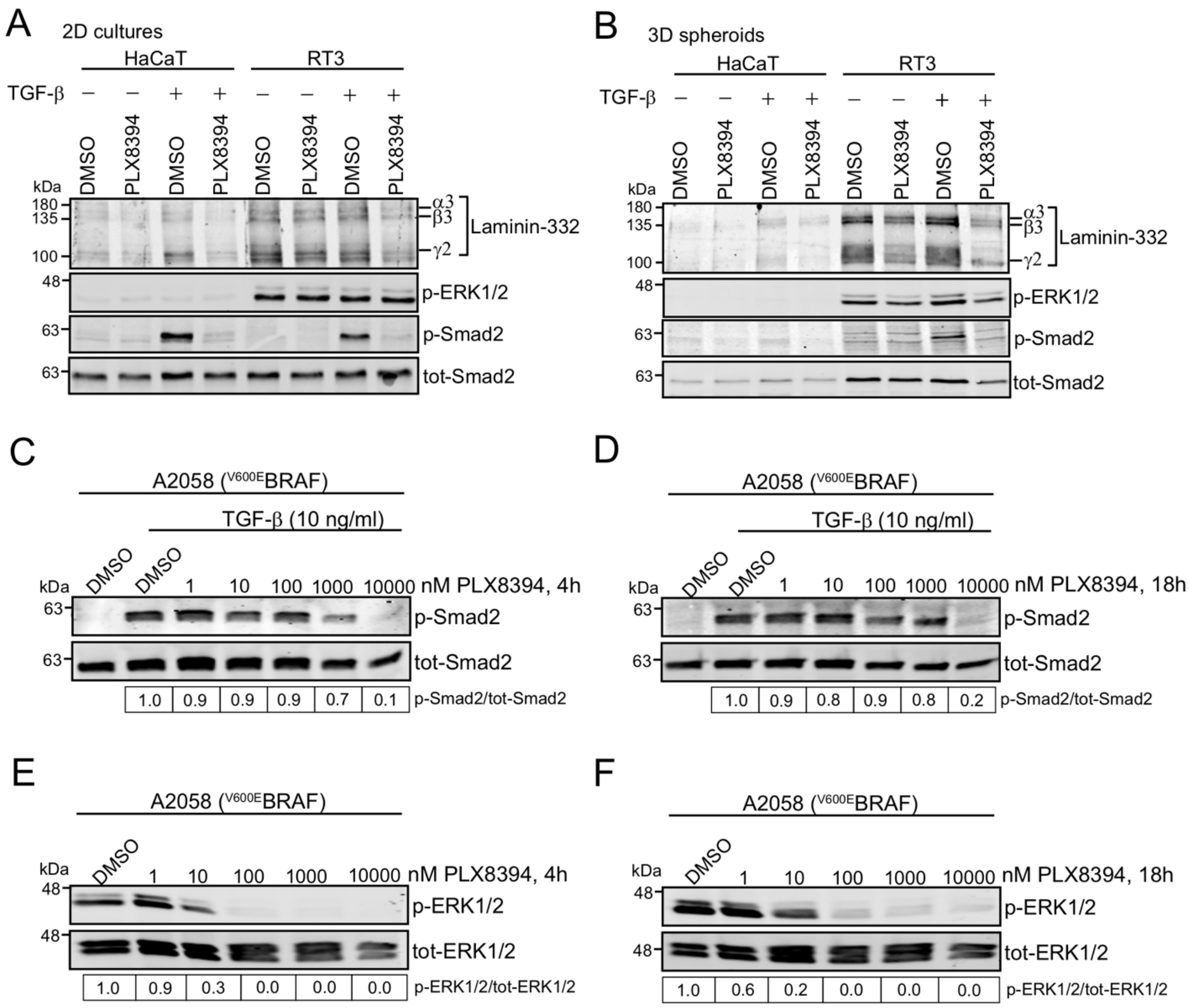

Supplementary Figure S1. **PLX8394 inhibits p-Smad2 and p-ERK1/2 synthesis, related to Figure 1.** (**A**) HaCaT and RT3 cells were treated with PLX8394 for 24 h in 2D cell culture condition. The cells were then subjected to TGF-β (10 ng/ml, 30 min) and harvested for western blotting. Laminin-332, p-ERK1/2, p-Smad2 and tot-Smad2 levels were analyzed by western blotting. (**B**) HaCaT and RT3 cells were first treated with PLX8394 in 2D cell culture condition for 24 h, followed by spheroid formation. Two-day-old spheroids were then subjected to TGF-β (10 ng/ml) for 4 h. Laminin-332, p-ERK1/2, p-Smad2 and tot-Smad2 levels were analyzed by western blotting. (**C and D**) A2058 melanoma cells were treated with increasing concentrations of PLX8394 for 4 h (C) or 18 h (D). The cells were then subjected to TGF-β (10 ng/ml, 30 min) and harvested for western blotting. p-Smad2 and tot-Smad2 levels were analyzed. Densitometric quantitation of p-Smad2 levels corrected for tot-Smad2 is shown below the blots. Values are relative to the levels (1.0) of DMSO plus TGF-β treated samples. (**E and F**) A2058 cells were treated with increasing concentrations of PLX8394 for 4

h (E) or 18 h (F). The cells were then harvested for western blotting and the levels of p-ERK1/2 and tot-ERK1/2 were analyzed. Densitometric quantitation of p-ERK1/2 levels corrected for tot-ERK1/2 is shown below the blots. Values are relative to the levels (1.0) of DMSO treated samples.

Supplementary Figure S2, related to Figure 2

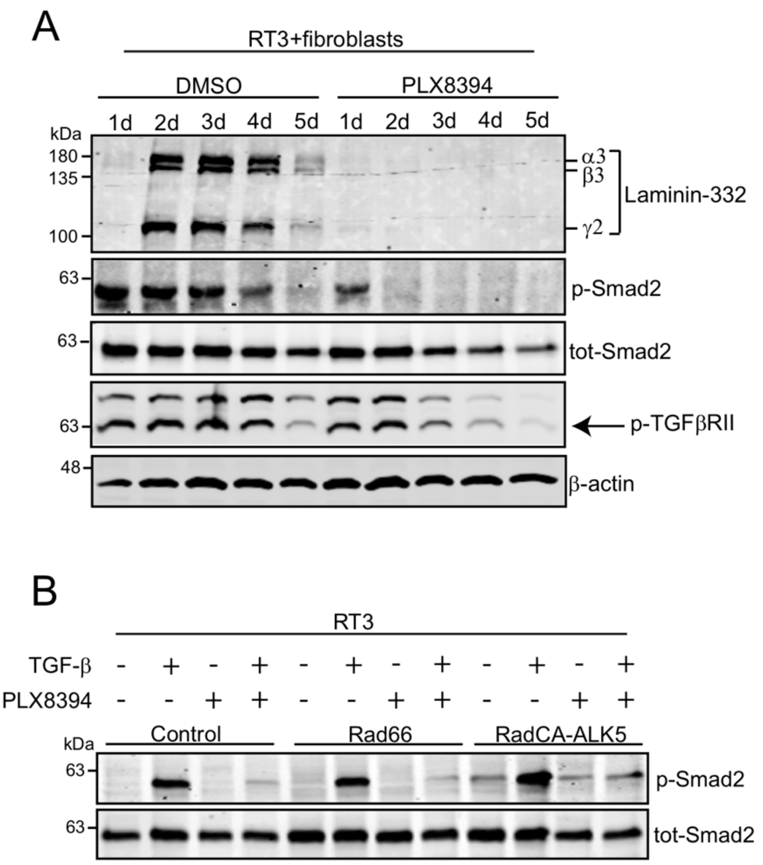

Supplementary Figure S2. PLX8394 inhibits TGF-β signaling by affecting TGF-β type II receptor kinase activity, related to Figure 2. (**A**) RT3 cells were treated with 10 μM PLX8394 for 24 h in 2D condition, followed by spheroid formation with skin primary fibroblasts. The spheroids were grown for one to five days. The levels of laminin-332, p-Smad2, tot-Smad2 and p-TGFβRII (arrow) were analyzed by western blotting. β-actin was used as a loading control. Representative images from three independent biological replicates are shown. (**B**) RT3 cells were either left uninfected or infected (100 MOI) with control virus RAd66 or with adenovirus coding for constitutively active ALK5 (RAdCA-ALK5) for 48 h. The cells were then treated with 10 μM PLX8394 for 24 h, followed by TGF-β treatment (10 ng/ml, 30 min). Phosphorylated Smad2 and tot-Smad2 levels were analyzed by western blotting. Representative images from four independent biological replicates are shown.

Supplementary Figure S3, related to Figure 3

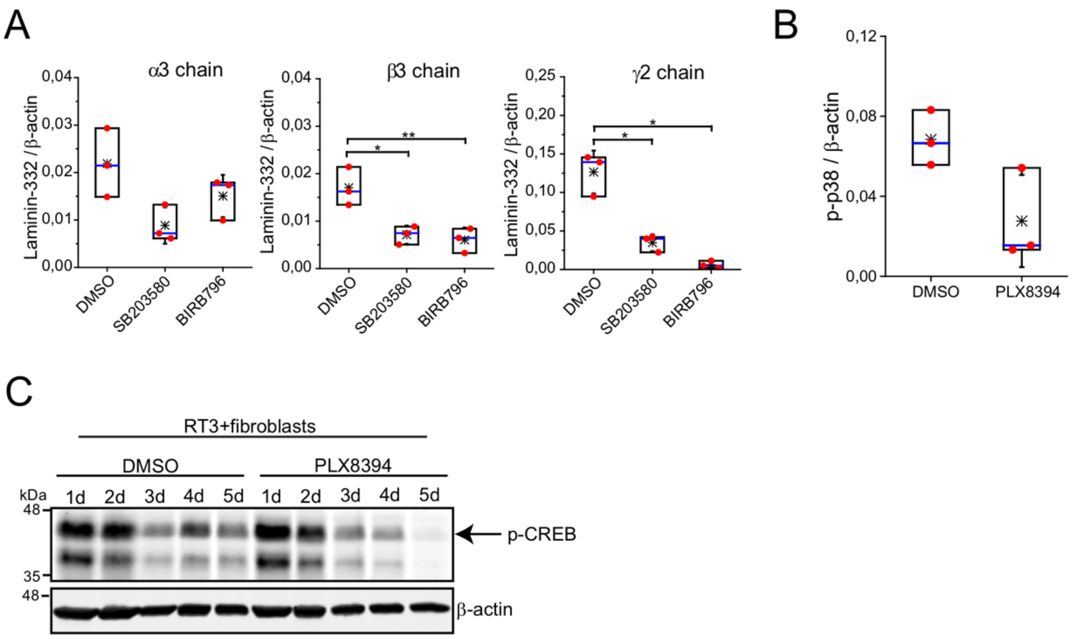

Supplementary Figure S3. p38 MAPK regulates laminin-332 expression in RT3 cells, related to Figure 3. (**A**) Quantification of laminin-332 α3, β3 and γ2 chain from western blots in Figure 3A. Box plots show data from three independent biological replicates (red dots), the second and third quartiles (the box), the median (blue line) and the mean (star) from all experiments ± S.D. $^{**}p<0.01$, $^{*}p<0.05$ (one-way ANOVA followed by Tukey or Dunnett's T3 post hoc tests). (**B**) Quantification of phosphorylated p38 from western blots in Figure 3B. Box plots show data from three independent biological replicates (red dots), the second and third quartiles (the box), the median (blue line) and the mean (star) from all experiments ± S.D. (**C**) RT3 cells were treated with 10 μM PLX8394 for 24 h in 2D condition, followed by spheroid formation with skin primary fibroblasts. The spheroids were grown for one to five days. The level of p-CREB was analyzed by western blotting. β-actin was used as a loading control. Representative images from three independent biological replicates are shown.

Supplementary Figure S4, related to Figure 4

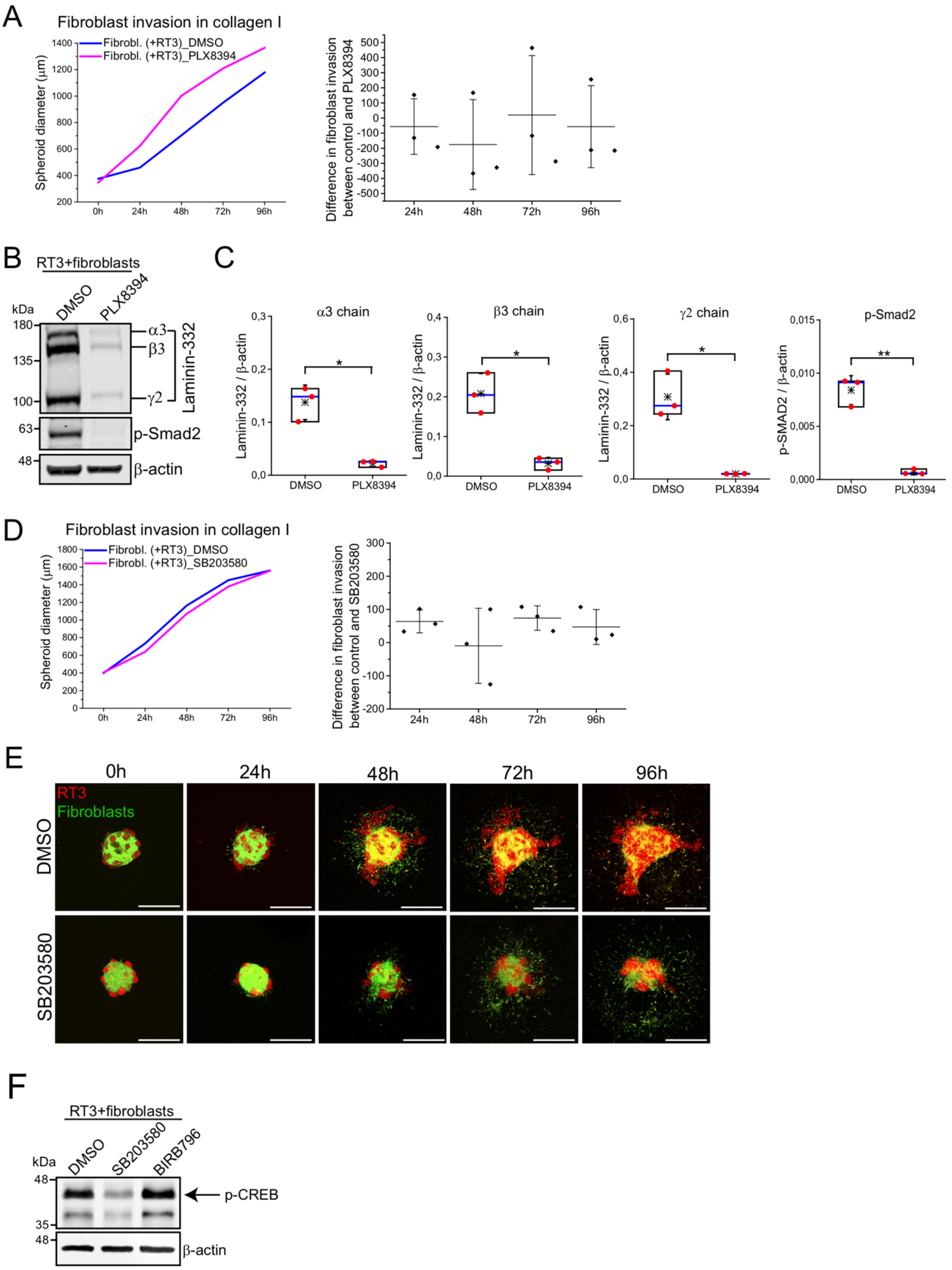

34

Supplementary Figure S4 (continued), related to Figure 4

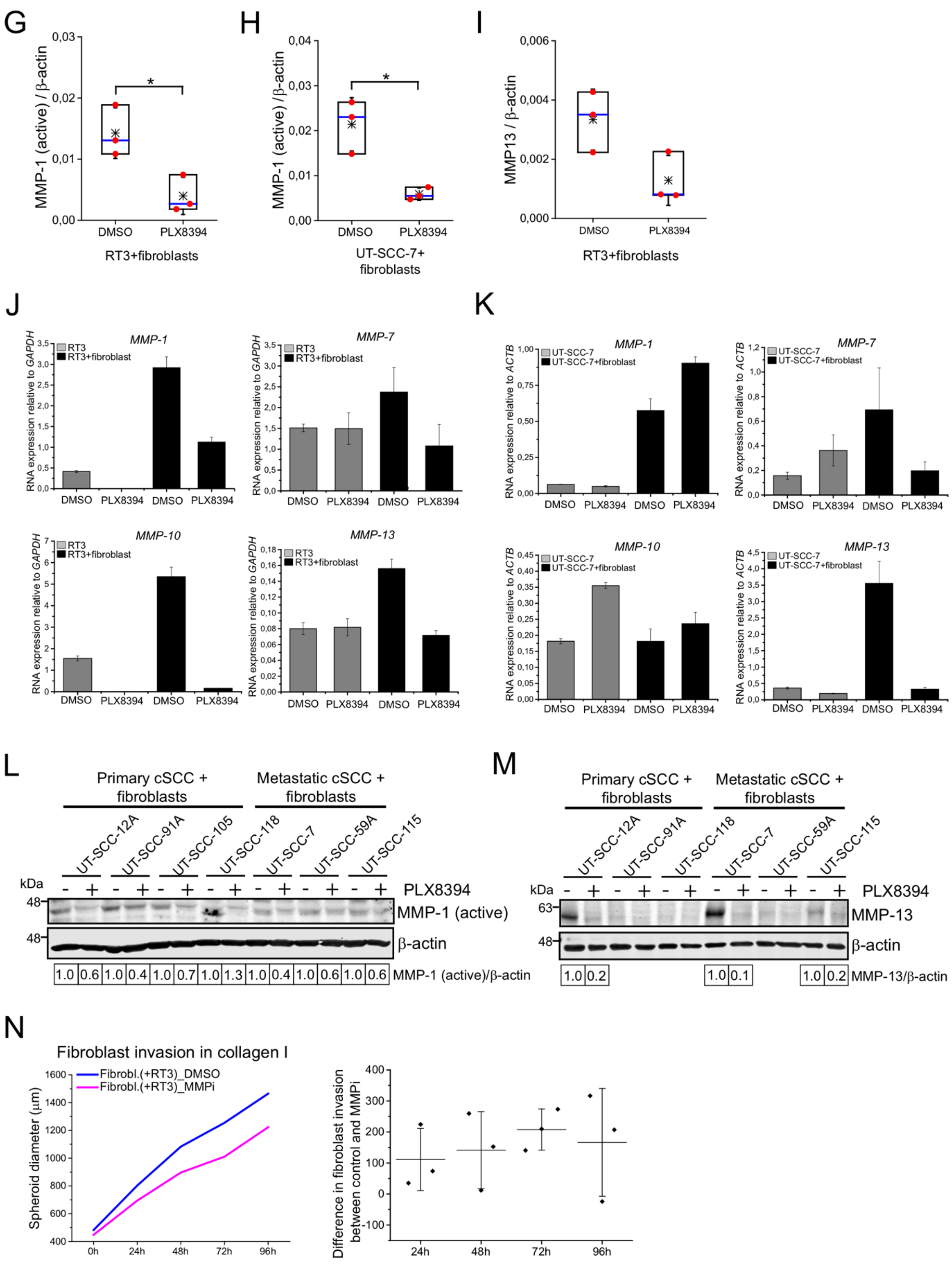

Supplementary Figure S4. PLX8394 inhibits RT3 cell invasion through collagen I, related to Figure 4.

(**A**) Fibroblast invasion from cocultured spheroids from Fig. 4B. *Left,* a representative graph from three biological replicates. *Right*, analysis of fibroblast invasion from cocultured spheroids. The graph shows difference in fibroblast invasion between DMSO-treated control samples and PLX8394-treated samples. The graph shows mean from three independent biological replicates (squares) ± SD. (Each replicate contained 3-4 spheroids). (**B**) Western blot analysis of spheroids used in the collagen invasion assay in Figs. 4A and 4B. RT3 cells were treated with 10 μM PLX8394 for 24 h, followed by spheroid formation with skin primary fibroblasts. The spheroids were then allowed to grow for three days and harvested for western blot analysis. Laminin-332 and p-Smad2 levels were analyzed by western blotting. β-actin was used as a loading control. Representative western blots from three independent biological replicates are shown. (**C**) Quantification of laminin-332 α3, β3 and γ2 chain levels and p-Smad2 from western blots in Figure S4B. Box plots show data from three independent biological replicates (red dots), the second and third quartiles (the box), the median (blue line) and the mean (star) from all experiments ± S.D. **p<0.01, *p<0.05 (Student's *t*-test). (**D**) Fibroblast invasion from cocultured spheroids from Fig. 4C. *Left,* a representative graph from three biological replicates. *Right*, analysis of fibroblast invasion from cocultured spheroids. The graph shows difference in fibroblast invasion between DMSO-treated control samples and SB203580-treated samples. The graph shows mean from three independent biological replicates (squares) ± SD. (Each replicate contained 3-4 spheroids). (**E**) Confocal images of spheroids used in the invasion assay in Fig. 4C. RT3 cells were treated with 10 μM SB203580 for 24 h in 2D condition, followed by spheroid formation with skin primary fibroblasts. The spheroids were allowed to grow for three days, after which they were transferred to a 96-well plate and embedded with a collagen I gel. The invasion was followed by a confocal microscope every 24 h during five days. Scale bar, 500 μm. From each time point, 2-4 spheroids were imaged and analyzed. Three independent biological replicates were performed. (**F**) Western blot images from the spheroids used in Fig. 4C. RT3 cells were treated with 10 μM SB203580 or 10 μM BIRB794 for 24 h in 2D condition, followed by spheroid formation with skin primary fibroblasts. The spheroids were allowed to grow for three days, after which they were harvested for western blotting and p-CREB (arrow) level was analyzed. β-actin was used as a loading control. (**G-I**) Quantification of MMP-1 (active form) (G and H) and MMP-13 (I) from western blots in Figs. 4D-F, respectively. Box plots show data from three independent biological replicates (red dots), the second and third quartiles (the box), the median (blue line) and the mean (star) from all experiments ± S.D. *p<0.05 (Student's *t*-test). (**J**) Expression of *MMP-1*, *MMP-7*, *MMP-10* and *MMP-13* in RT3 spheroids cultured with (black bars) or without (grey bars) human skin primary fibroblasts. Expression was measured using real-time quantitative PCR and

corrected for the levels of *GAPDH* mRNA. (**K**) Expression of *MMP-1*, *MMP-7*, *MMP-10* and *MMP-13* in cSCC spheroids cultured with (black bars) or without (grey bars) human skin primary fibroblasts. Expression was measured using real-time quantitative PCR and corrected for the levels of *ACTB* (β-actin) mRNA. (**L**) A panel of primary (SCC-12A, SCC-91A, SCC-105, SCC-118) and metastatic (SCC-7, SCC-59A, SCC-115) cSCC cell lines were treated with 10 μM PLX8394 for 24 h in 2D condition, followed by spheroid formation with skin primary fibroblasts. The spheroids were grown three days. The levels of MMP-1 was analyzed by western blotting and β-actin was used as a loading control. Densitometric quantitation of MMP-1 levels corrected for β-actin is shown below the blots. Values are relative to the levels (1.0) of the control sample (without PLX8394) of each cell line. MMP-1 was detected from the same membrane as in Fig. 1C and thus the same β-actin blot is shown. (**M**) MMP-13 was detected by western blotting in a panel of primary and metastatic cSCC cell lines. The cells were treated with 10 μM PLX8394 for 24 h in 2D condition, followed by spheroid formation with skin primary fibroblasts. The spheroids were grown three days. The levels of MMP-13 was analyzed by western blotting and β-actin was used as a loading control. Densitometric quantitation of MMP-13 levels corrected for β-actin is shown below the blots. Values are relative to the levels (1.0) of control sample (without PLX8394) of the selected cell lines. (**N**) Fibroblast invasion from cocultured spheroids from Fig. 4G. *Left,* a representative graph from three biological replicates. *Right*, analysis of fibroblast invasion from cocultured spheroids. The graph shows difference in fibroblast invasion between DMSO-treated control samples and MMP inhibitor -treated samples. The graph shows mean from three independent biological replicates (squares) ± SD. (Each replicate contained 5-8 spheroids).

Supplementary Figure S5, related to Figure 5

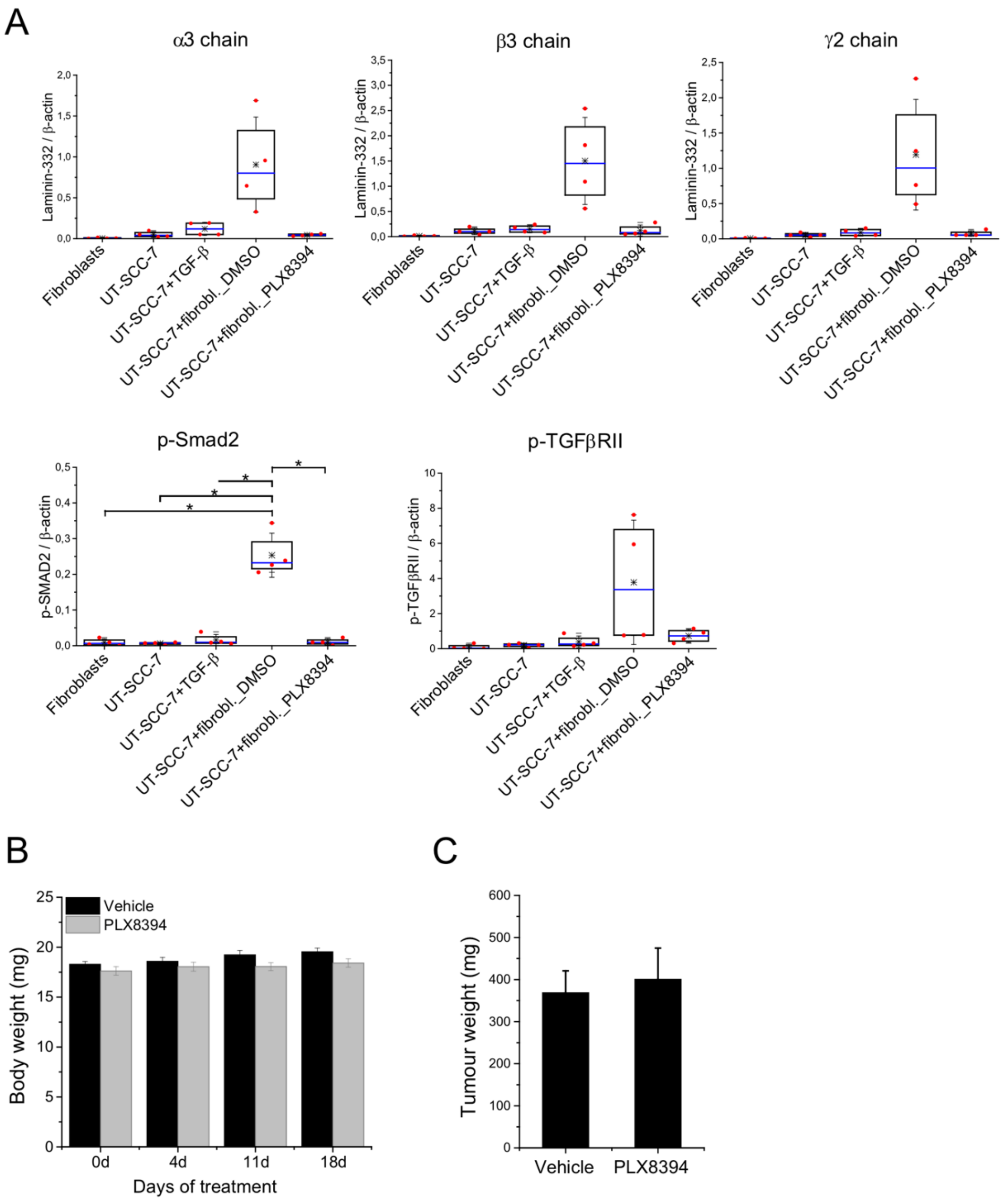

Supplementary Figure S5. PLX8394 inhibits laminin-332 production, growth and invasion of human cSCC xenografts *in vivo*, related to Figure 5. (**A**) Quantification of laminin-332 α3, β3 and γ2 chain, p-Smad2 and p-TGFβRII from western blots in Figure 5A. Box plots show data from three independent biological replicates (red dots), the second and third quartiles (the box), the median (blue line) and the mean (star) from all experiments ± S.D. *p<0.05 (one-way ANOVA followed by Dunnett's T3 post hoc test). (**B**) Weights derived from mice treated with vehicle or PLX8394 (150 mg/kg, once daily) 0, 4, 11 and 18 days after oral administration. (**C**) Tumor weights from mice treated with vehicle or PLX8394 (150 mg/kg, once daily) for 18 days.

Supplementary Table S1. Identified matrisome proteins in the laser capture microdissected human cSCC xenograft.

Supplementary Table 1. Identified matrisome proteins in the laser capture microdissected human cSCC xenograft.

Protein names	Gene symbol	Peptides unique to mouse	Peptides unique to human	Peptides common to both	Species[a]
Adiponectin	ADIPOQ	2	0	0	Mouse
Alpha-1-antitrypsin 1-2	SERPINA1B	3	0	0	Mouse
Alpha-1-antitrypsin 1-4	SERPINA1D	3	0	0	Mouse
Alpha-2-antiplasmin	SERPINF2	2	0	0	Mouse
Alpha-2-macroglobulin	A2M	13	0	0	Mouse
Alpha-2-macroglobulin-like protein 1	A2ML1	0	16	0	Human
Annexin A1	ANXA1	0	9	7	Human
Annexin A2	ANXA2	1	8	18	Human
Annexin A3	ANXA3	0	5	0	Human
Annexin A4	ANXA4	0	8	4	Human
Annexin A5	ANXA5	4	6	5	Mouse and Human
Annexin A7	ANXA7	0	1	8	Ambiguous
Annexin A8	ANXA8	0	1	4	Ambiguous
Antithrombin-III	SERPINC1	8	0	0	Mouse
Biglycan	BGN	1	0	3	Ambiguous
Cathepsin B	CTSB	1	3	0	Human
Cathepsin D	CTSD	0	13	1	Human
Cathepsin L2	CTSV	0	5	0	Human
CD109 antigen	CD109	0	3	0	Human
Collagen alpha-1(I) chain	COL1A1	23	0	20	Mouse
Collagen alpha-1(III) chain	COL3A1	29	0	7	Mouse
Collagen alpha-1(V) chain	COL5A1	0	0	2	Ambiguous
Collagen alpha-1(VI) chain	COL6A1	12	0	6	Mouse
Collagen alpha-1(VII) chain	COL7A1	28	4	8	Mouse and Human
Collagen alpha-1(XII) chain	COL12A1	29	4	30	Mouse and Human
Collagen alpha-1(XVII) chain	COL17A1	0	6	2	Human
Collagen alpha-2(I) chain	COL1A2	36	0	12	Mouse
Collagen alpha-2(VI) chain	COL6A2	9	0	8	Mouse
Collagen alpha-3(VI) chain	COL6A3	53	1	14	Mouse
Cystatin-A	CSTA	0	5	0	Human
Cystatin-B	CSTB	0	3	0	Human
Elafin	PI3	0	5	0	Human
Extracellular matrix protein 1	ECM1	0	4	0	Human
Fibrillin-1	FBN1	7	1	15	Mouse
Fibrinogen alpha chain	FGA	8	0	0	Mouse
Fibrinogen beta chain	FGB	5	0	4	Mouse
Fibrinogen gamma chain	FGG	7	0	3	Mouse
Fibronectin	FN1	7	0	5	Mouse
Fibulin-2	FBLN2	3	0	0	Mouse
Galectin-1	LGALS1	1	0	2	Ambiguous
Galectin-3	LGALS3	0	4	1	Human
Galectin-7	LGALS7	1	10	0	Human
Hemopexin	HPX	13	0	1	Mouse
Inter alpha-trypsin inhibitor, heavy chain 4	ITIH4	1	0	1	Ambiguous
Kininogen-1	KNG1	5	0	0	Mouse
Laminin subunit beta-3	LAMB3	0	7	0	Human

Supplementary Table S2. List of specific primers and probes for real-time quantitative PCR.

Supplementary Table 2.

List of specific primers and probes for real-time quantitative PCR.

Gene/RNA		Sequence
MMP1	Forward	5′-AAGATGAAACGTGGACCAACAATT-3′
	Reverse	5′-CCAAGAGAATGGAAGAGTTC-3′
	Probe	5′-FAM-CAGAGAGTACAACTTACATCGTGTTGCGGCTC-TAMRA-3′
MMP7	Forward	5′-CTTTGCGCGAGGAGCTCA –3′
	Reverse	5′-CAGGCGCAAAGGCATGA –3′
	Probe	5′-FAM-CCATTTGATGGGCCAGGAAACACG-TAMRA–3′
MMP10	Forward	5′-GGACCTGGGCTTTATGGAGATAT-3′
	Reverse	5′-CCCAGGGAGTGGCCAAGT-3′
	Probe	5′-FAM-CATCAGGCACCAATTTATTCCTCGTTGCT-TAMRA-3′
MMP13	Forward	5′-AAATTATGGAGGAGATGCCCATT-3′
	Reverse	5′-TCCTTGGAGTGGTCAAGACCTAA-3′
	Probe	5′-FAM-CTACAACTTGTTTCTTGTTGCTGCGCATGA-TAMRA-3′
ACTB	Forward	5′-TCACCCACACTGTGCCCATCTACGC-3′
	Reverse	5′-CAGCGGAACCGCTCATTGCCAATGG-3′
	Probe	5′-FAM-CAGCGGAACCGCTCATTGCCAATGG-BHQ1-3′
GAPDH	Forward	5'-ACCCACTCCTCCACCTTTGA-3'
	Reverse	5'-TTGCTGTAGCCAAATTCGTTGT-3'
	Probe	5'-FAM-ACGACCACTTTGTCAAGCTCATTTCCTGGT-BHQ1-3'
